# Space Use of an Expanding Generalist Predator Is Shaped by Human, Marine and Seasonal Effects on Arctic Tundra

**DOI:** 10.1002/ece3.72512

**Published:** 2025-11-17

**Authors:** Stijn P. Hofhuis, Arnaud Tarroux, Rolf A. Ims, Dorothee Ehrich

**Affiliations:** ^1^ Department of Arctic and Marine Biology UiT—The Arctic University of Norway Tromsø Norway; ^2^ Norwegian Institute for Nature Research Tromsø Norway

**Keywords:** coast, habitat selection, home range, movement, red fox, *Vulpes vulpes*

## Abstract

Generalist species that benefit from human impacts on terrestrial ecosystems and expand their distribution range can threaten biodiversity by outcompeting or predating on native specialists. This is exemplified by the northward expansion of the red fox onto the arctic tundra where this mesopredator threatens endemic arctic fox and ground‐nesting bird populations. Here, effective management efforts to control the expansion depend on understanding the spatiotemporal scales of red fox movement within the tundra, and on identifying habitats that provide food resources to red foxes. We addressed these needs by studying the geographic space use and habitat selection of 14 red foxes on the low Arctic tundra of Varanger Peninsula in Norway by means of GPS telemetry. Red foxes used large home ranges and were especially mobile during winter, partly owing to occasional movements beyond home range boundaries. Home ranges were significantly smaller near the marine coastline and at higher human land use intensities, likely owing to higher food availability. These habitat features were also selected for within individual home ranges, confirming the importance of these habitats to red foxes on the tundra. High mobility necessitates large‐scale culling efforts aimed at achieving long‐term reductions in red fox densities. However, localised and temporary effects may be achieved by aligning culling with periods of reduced red fox movement and breeding of vulnerable species in late spring and summer. Additionally, reducing food subsidies associated with human land use could mitigate the underlying drivers of red fox expansion, offering a more sustainable approach to management.

## Graphical abstract

Red foxes that expand into tundra ecosystems threaten biodiversity, and knowledge on their space use is needed to inform management efforts. We found that red fox space use varied with seasons and with environmental contexts. Space use is likely affected by food densities linked to seasonality, human land use and the marine coastline.

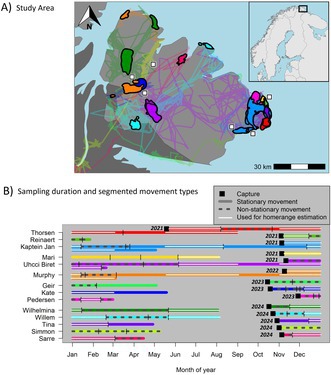

## 1 Introduction

Increased human impacts on terrestrial ecosystems (Venter et al. 2016) and climate change have caused rapid shifts in species' distribution ranges in recent decades (Chen et al. 2011; Pacifici et al. 2020). Generalist species with broad ecological niches and superior competitive or dispersal abilities tend to take advantage of increased food resource availability associated with human land use and climate warming, allowing them to expand their distribution ranges (Sweeney and Jarzyna 2022; Urban et al. 2012). Expanding generalists can negatively impact biodiversity when they outcompete or prey on native specialists that are maladapted to these interactions (Clavel et al. 2011; Urban et al. 2012). Ecosystems located at ecotone edges, where interactions between dissimilar species are more likely, and ecosystems with a simple trophic structure and interspecific interactions that are consequently more tightly coupled, are particularly vulnerable (Roemer et al. 2009; Wallingford et al. 2020). Management efforts that attempt to mitigate the negative impacts of expanding generalists depend on understanding their environmental drivers and the spatiotemporal scales at which management interventions should be applied.

Arctic tundra ecosystems near the forest–tundra ecotone exemplify the issues of expanding generalists. Tundra ecosystems are characterised by relatively simple vertebrate food webs with few species, low ecosystem productivity and high temporal fluctuations in the availability of food resources (Ims and Fuglei 2005; Krebs et al. 2003). During winter, resources are scarce or inaccessible due to snow cover, while short summers produce a temporary abundance that attracts migratory species (Callaghan et al. 2004; Gauthier 2004; Lindström et al. 1994). Food resource availability to predators also varies greatly from year to year, particularly due to 3–5‐year population cycles of small rodents (lemmings and voles), which are important herbivores and prey within the food web (Ims and Fuglei 2005). Endemic tundra specialist species like the arctic fox (*Vulpes lagopus*) are therefore adapted to survive long periods of food scarcity (Callaghan et al. 2004). Currently however, rapid climate warming and increased human land use provide increased food resources within the tundra, likely supporting the northward expansion of southern generalist species that were previously food‐limited (Elmhagen et al. 2015). Among the most disruptive of these species are red foxes (*Vulpes vulpes*), which over the last century have sustained a considerable northward expansion on both the Eurasian and North American continents (Elmhagen et al. 2017; Gallant et al. 2020; Hersteinsson and MacDonald 1992).

Red foxes in Fennoscandian tundra ecosystems outcompete and prey on threatened endemic populations of arctic foxes and ground‐nesting birds, including attractive game species such as ptarmigan (Elmhagen et al. 2017; Henden et al. 2021; Marolla et al. 2019). To protect these species, conservation efforts often apply red fox culling (Angerbjörn et al. 2013). However, the effect of culling is not always clear (Marolla et al. 2019). This is partly because red foxes are highly mobile, and culling must therefore be applied at the appropriate spatial scales and times of year (Kämmerle and Storch 2019; Lieury et al. 2015). Determining which spatiotemporal scales are suitable for effective management requires sound knowledge of the red foxes' geographic space use patterns, such as seasonal home range sizes and displacement distances over time (Carter et al. 2012; Fraser et al. 2018).

Individuals at the periphery of a species' distribution range tend to use larger home ranges due to increased scarcity in food resources (Linnell et al. 2021; Niedzielski and Bowman 2016), as predicted by the resource dispersion hypothesis (Macdonald 1983). Indeed, red foxes in tundra ecosystems have some of the largest reported home range sizes (Lai et al. 2022; Warret Rodrigues and Roth 2023). These studies, however, were conducted within uninhabited and relatively uniform tundra habitats. Little is known about the effects of spatial gradients in human land use and natural environmental productivity on red fox home range size within the tundra. Such knowledge is important given increased human land use and rapid climate warming in the Arctic. Moreover, seasonal variation in food resource availability may lead to corresponding changes in home range size. Animals may either adopt a ‘flexible strategy’ by expanding home ranges during periods of scarcity or maintain stable home ranges large enough to meet minimal needs (Von Schantz 1984). Both strategies have been observed in red foxes, including those in tundra ecosystems (Lai et al. 2022; Meia and Weber 1995; Warret Rodrigues and Roth 2023). Limited consensus therefore exists about variation in red fox home range size throughout the annual cycle on the tundra. Beyond moving within home ranges, red foxes may also undertake exploratory excursions to access locally concentrated food resources (Carter et al. 2012; Tsukada 1997), to reproduce or disperse (Oehler et al. 2025; Soulsbury et al. 2011; Walton et al. 2018). The timing and extent of these movements in geographic space are also likely dependent on seasonal variation in resource densities and breeding phenology (Lai et al. 2022; Soulsbury et al. 2011; Tsukada 1997; Walton et al. 2018).

Though current management interventions in Fennoscandia focus on culling, effective management actions should arguably target the environmental drivers originally enabling red fox expansion. Generalist species in peripheral populations likely face increased environmental selection pressures because they operate at the margins of their environmental tolerance (Prieto‐Ramirez et al. 2018). Studying habitat selection in peripheral conditions may therefore help identify these selection pressures. The concept of hierarchical scaled habitat selection (Johnson 1980) has helped to identify red fox selection for both human—and naturally productive habitats and multiple spatial scales. At the scale of the red fox's geographic range (first order selection; Johnson 1980), the northern distribution limit is likely determined by human presence and winter severity (Bartoń and Zalewski 2007; Gallant et al. 2020). At landscape scale (second order selection), tundra red foxes are known to be more abundant within naturally productive lowlands (Frafjord 2003; Killengreen et al. 2007; Stoessel et al. 2019) and in areas with more human land use seen in terms of infrastructure and semi‐domestic reindeer herding (Henden et al. 2014; Rød‐Eriksen et al. 2020; Stickney et al. 2014). Diet (fourth order selection) studies have further confirmed that food items from both human sources and naturally productive sources—including both marine and terrestrial sources—are important (Killengreen et al. 2011; Savory et al. 2014). Less is known however, about how selection for human and naturally productive tundra environments extends to habitat selection within individual home ranges (third order selection). This is important, because habitats at this scale provide practical targets for strategies that aim to reduce resource availability (White et al. 2006).

The Arctic tundra of Varanger Peninsula in Norway lies just north of the forest–tundra ecotone and provides an excellent setting for studying these questions. Here, red foxes have increased in abundance over the last century (Ims et al. 2017; Johnsen 1929), and are currently widespread despite intensive culling (Killengreen et al. 2012). Steep spatial gradients in human land use and natural environmental productivity on Varanger Peninsula provide an opportunity to study their effect on geographic space use and third order habitat selection of red foxes in a tundra ecosystem. Using GPS telemetry on 14 red foxes throughout the annual cycle, we calculated seasonal (summer and winter) home range sizes and maximum net displacements on weekly and monthly timescales. We then examined how seasonal home range sizes varied with the degree of human land use and natural environmental productivity within the home range. We additionally studied habitat selection within individual home ranges by means of resource selection functions. We expected smaller home ranges during summer, and in areas closer to the coast, with higher human land‐use intensity, and higher vegetation productivity, as these conditions likely correspond to higher food resource densities, requiring smaller home ranges to ensure sufficient food security (Macdonald 1983). We also expected foxes to select for habitats closer to the coast, with higher land‐use intensities, and higher vegetation productivity within individual home ranges for the same reason. Finally, we expected greater maximum net displacements during winter, reflecting a need to access scarce resources and an increase in exploratory excursions associated with dispersal and mating, both of which tend to occur during winter.

## 2 Methods

### 2.1 Study Area

**FIGURE 1:**
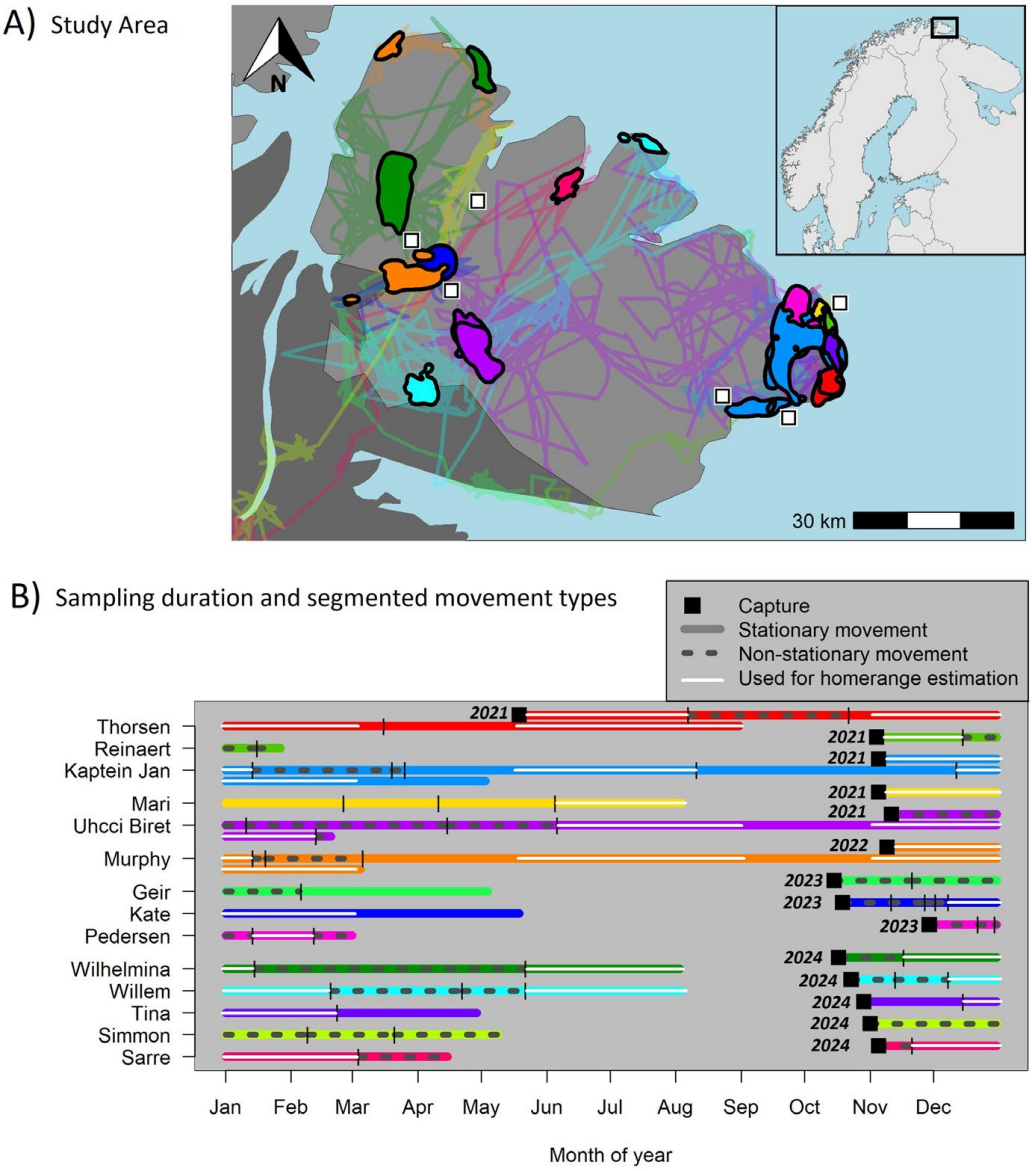
(A) Study area with fox movements and home ranges. Lines indicate movement trajectories, while polygons indicate estimated home ranges. White squares indicate approximate trapping locations. Light grey indicates the extent of low Arctic tundra, dark grey indicates mountain birch forest (Pedersen et al. 2021). (B) Sampling duration and segmented movement phases over an annual cycle. Vertical black lines indicate breakpoints in movement phases. Colours represent the same individual foxes in both panels.

This study was conducted during 2021–2025 on the ca. 6000 km^2^ Varanger Peninsula, Finnmark (hereafter Varanger) located at the northeastern tip of Norway (70°–71° N 28°–31° E) (Figure 1A). The interior and northern and eastern lowlands on the peninsula (ca. ¾ of the total area) are classified as low Arctic tundra (Pedersen et al. 2021, Figure 1A), with mean July temperatures below 10°C during 1960–1990 (Hanssen‐Bauer and Tveito 2014; Ims et al. 2013). The most extensive tundra plant community is dwarf shrub heath (Killengreen et al. 2007), but topographic and climatic gradients lead to a mosaic of tundra vegetation types (Ravolainen et al. 2010) ranging from low productive barrens in the inland highlands (350–600 m asl) (Ims et al. 2013), to thickets of tall willow shrubs and lush meadow vegetation in riparian valleys (Ravolainen et al. 2013). The southwestern lowlands have higher mean July temperatures (11°C–13°C, 1960–1990) and are forested by mountain birch ( *Betula pubescens* ) (Ims et al. 2017) (Figure 1A). Owing to the surrounding ice‐free Barents Sea and the North Atlantic current, winters are relatively mild for this latitude. January temperatures average −6°C and snow cover typically lasts from November through April/May (Norwegian Centre for Climate Services 2025), however, there is a steep gradient from the relatively colder inland to the milder coast (Ims et al. 2013). Human population density on Varanger is around 2 persons/km^2^ (Statistics Norway 2025), the vast majority of which is concentrated in settlements along the coast. Other important types of human land use include reindeer ( *Rangifer tarandus* ) herding, livestock farming, a marine fishing industry and tourism.

More than 4500 red foxes have been culled on Varanger over the last 20 years as one of several measures to conserve an endangered population of arctic foxes. While arctic foxes used to be the numerically dominant fox species, less than 50 individuals currently remain (Eide et al. 2024). Arctic foxes use breeding dens in the remote, least productive, inner parts of the peninsula in spring and summer (Ehrich, Knutsen, et al. 2025; Killengreen et al. 2007). In contrast, red fox culling occurs mainly in winter and along the human‐inhabited, more productive coastline.

The terrestrial ecosystem, the surrounding marine ecosystem, and human land use, each supply potentially important food resources to red foxes on Varanger. Small rodents are the most important natural prey (Killengreen et al. 2011), and three species are of functional importance: the grey‐sided vole ( *Myodes rufocanus* ), the tundra vole ( *Microtus oeconomus* ) and the Norwegian lemming ( *Lemmus lemmus* ) (Ims et al. 2017). These species have spatially and temporally synchronous population cycles with peak abundances every 4 to 5 years (Ims et al. 2011, 2017). Peak abundances occurred during the study period in 2022–23 (Ehrich, Knutsen, et al. 2025). Other terrestrial prey observed in stomach contents include hare ( *Lepus timidus* ), ptarmigan ( *Lagopus lagopus* or *Lagopus muta* ) and other birds (Ehrich, Knutsen, et al. 2025). Food items of marine origin (including fish, invertebrates or sea birds) are known to be important to red foxes in the study area during winters when small rodents are scarce (Killengreen et al. 2011). Reindeer carcasses are also known to constitute an important food subsidy on Varanger (Killengreen et al. 2011), but little is known about the importance of other human‐derived food items.

Red foxes have few natural predators on Varanger. Wolves are absent, wolverines ( *Gulo gulo* ) are limited in abundance, but commonly occurring white‐tailed eagles ( *Haliaeetus albicilla* ) and golden eagles ( *Aquila chrysaetos* ) may take pups.

The timing of red fox breeding phenology is known to be delayed at higher latitudes (Lloyd 1981). Demographic data from our study area indicate that reproduction occurs during February and gestation from mid‐February to mid‐May (Ehrich, Kaino, et al. 2025). Previous research suggests that red fox dispersal primarily occurs between September and February (Jensen 1973; Lloyd 1981; Storm et al. 1976) but may occur until June (Walton et al. 2018; Warret Rodrigues and Roth 2023).

### 2.2 Environmental Variables

We used three environmental variables that represent the main spatial gradients in likely food resource availability to red foxes in our study area: (1) Enhanced Vegetation Index (EVI), (2) the natural logarithm of the distance to coastline (LogCoastDist) and (3) human land use intensity (LUI). These three variables are discussed in more detail below. All data processing, spatial and statistical analyses were performed using R software (R Core Team 2023).

EVI: Productive tundra vegetation types like meadows and willow thickets are known to be important habitat for numerous potential prey species, including tundra voles, willow ptarmigan and hares (Ehrich et al. 2012; Henden et al. 2011). Vegetation productivity was expressed using Enhanced Vegetation Index (EVI), derived from high‐quality remote sensing data from the MODIS platform (Didan 2021) by Tveraa et al. (2013). EVI values below 0.1 were set to NA in this data set. For each pixel, we calculated the mean of maximum yearly EVI values from 2000 to 2021 from a minimum of 10 non‐NA values. To address erroneous low EVI values (set to NA) at the coastline, caused by pixels overlapping with water, we interpolated NA values using a 5‐pixel moving window (focal function, terra package) (Hijmans et al. 2025). Finally, all remaining NA values were reset to the minimum EVI value of 0.1.

LogCoastDist: The marine coastline provides access to seabirds, marine invertebrates or other food resources like carrion that may drift onshore. To represent a gradient of increased availability of these resources at closer distances to the coast, we used the natural logarithm of the distance to the coastline with a minimum distance of 100 m. Coastline data were obtained from OpenStreetMap (OpenStreetMap contributors 2024), and distances were rasterised using sf and terra packages (Hijmans et al. 2025; Pebesma et al. 2025).

LUI: Human land use is known to generate food subsidies from waste, intentional feeding or wildlife collisions with infrastructure (Newsome et al. 2015; Oro et al. 2013). Human land‐use intensity data were retrieved in raster format from Artsdatabanken (2019) and represents an index of human influence on the landscape measured in terms of infrastructure density on a continuous scale from 0 (indicating no influence) to 260 (indicating urbanised city centres).

All three environmental layers were reprojected (project function) to WGS 84/UTM Zone 35 N and aligned to the extent and resolution of the coarsest layer (EVI, 182 m) (resample function) using the terra package (Hijmans et al. 2025). Reprojecting the EVI data layer resulted in a change in resolution from the original MODIS data of 250 m (Sinusoidal projection) to 182 m. Original projections of LUI and coastline data were Web Mercator and WGS 84, respectively. We excluded areas that were considered inaccessible to foxes by assigning NA to pixels beyond 50 m of the marine coastline and pixels overlapping more than 99% with lakes. Lakes were mapped using data from the Norwegian Environment Agency (2023).

### 2.3 GPS Telemetry

Between 2021 and 2025, 14 red foxes (Table 1) were captured in large (3–4 m^2^) wooden double‐entry traps and equipped with Iridium Litetrack 150 collars weighing 170 g (Lotek 2025). Foxes were detained within the trap for a maximum of 12 h and handled for 30 min on average before release. No chemical immobilisation was used. Capture, handling and collar deployment were in accordance with animal experiment procedures approved by the Norwegian Food Safety Authority (FOTS ID 30085).

**TABLE 1:** Summary information of GPS tracking data and individual characteristics of foxes included in the study.

Animal ID	Life stage at capture	Sex	Tracking duration first‐last position (days)	Total number of positions	Longest gap between positions/*within home ranging periods* (days)		Total number of days without positions/*within home ranging periods*		Maximum net displacement over tracking duration (km)
Thorsen	Adult	♂	469	4225	1.9	*1.9*	17	*16*	27
Reinaert	Subadult	♂	84	758	1.0	*1.0*	0	*0*	65
Kaptein Jan	Subadult	♂	545	5218	2.0	*0.9*	2	*0*	31
Mari	Subadult	♀	272	2320	1.3	*1.3*	3	*2*	8
Uhcci Biret	Subadult	♀	467	4650	1.4	*1.3*	3	*1*	79
Murphy	Subadult	♂	512	3768	17.7	*9.0*	116	*73*	54
Geir	Subadult	♂	203	1645	1.5	*NA*	4	*NA*	57
Kate	Adult	♀	213	800	20.1	*9.8*	111	*46*	27
Pedersen	Subadult	♀	93	673	1.0	*0.9*	0	*0*	21
Wilhelmina	Adult	♀	290	2226	9.3	*9.3*	52	*52*	48
Willem	Subadult	♂	285	2437	5.3	*5.3*	13	*13*	106
Tina	Subadult	♀	182	587	15.0	*1.5*	29	*4*	16
Simmon	Subadult	♂	190	631	5.0	*NA*	24	*NA*	116
Sarre	Subadult	♂	161	1384	1.1	*0.9*	1	*0*	337

Tracking duration ranged between 84 and 512 days (Table 1). Collars were attached permanently, and the carcasses of seven collared foxes were retrieved after being shot by hunters or hit by a vehicle. We conducted autopsies (Ehrich 2025), which showed these foxes to be in good health. No weight loss was observed, and placental scars indicated that two females had reproduced.

Collars were programmed to record GPS fixes every 3 h. An additional GPS schedule recorded fixes every 20 min during 3 h at night (covering periods between 15:00 and 6:00 GMT) every other day. Collars were remotely reprogrammed to record 1 or 2 fixes per day upon low battery or transmission problems. Transmission problems led to multiple gaps of 2–20 days between successive locations in seven collars. Days without GPS positions represented up to 50% of the total tracking duration in the most extreme case (Table 1).

**TABLE 2:** Effects of predictors on the natural logarithm of the home range size. Fixed effects are presented with 95% confidence intervals (in parentheses).

Statistical method	Predictor variables	Effects	Sample size
Paired *t*‐test	Season	**0.314** (0.015, 0.612)	5 pairs
Linear mixed‐effects model	Season LogDistCoast Individual (random)	**0.516** (0.186, 0.824) **0.411** (0.312, 0.505) Variance = 0.165	22 home ranges 12 individuals
Linear mixed‐effects model	Season Mean LUI Individual (random)	0.282 (−0.263, 0.834) **−0.018** (−0.028, −0.009) Variance = 0.141	22 home ranges 12 individuals
Linear mixed‐effects model	Season Mean EVI Individual (random)	0.101 (−0.617, 0.842) −0.103 (−6.491, 6.351) Variance = 0.168	22 home ranges 12 individuals

*Note:* Statistically significant fixed effects (i.e., 95% CI not overlapping 0) are in bold. Positive season effects imply larger winter than summer home ranges. LogDistCoast, the natural logarithm of the distance to the coastline.

Abbreviations: EVI, enhanced vegetation index; LUI, human land use intensity.

GPS locations were reprojected from the geographic coordinate system WGS 84 to the projected coordinate system WGS 84/UTM zone 35 N throughout the analysis. GPS accuracy was assessed using calibration data from two stationary collars with a 1‐h fix interval (*n* = 19 fixes). The root‐mean‐square User Equivalent Range Error (UERE), estimated using the ueri.fit function in the ctmm package (Fleming et al. 2025), was 2.24 to 3.63 m. Because this error is negligible compared to the spatial resolution of our environmental raster data (182 m), we concluded that GPS error would not significantly affect our analyses. We used the outlie function in the ctmm package to identify and remove four positional outliers based on a maximum speed threshold of 30 km/h. After filtering, the maximum observed speed was below 10 km/h.

### 2.4 Statistical Analysis

#### 2.4.1 Geographic Space Use

We described the geographic space use of individual foxes over time using two methods.

1. Seasonal home range sizes: Home ranges were estimated separately for summer (May 15th—September 15th) and winter (November 1st—March 1st) based on periods when foxes exhibited stationary movement behaviour, thereby excluding dispersal, exploratory or nomadic movements. To identify and segment stationary and nonstationary movement phases we first thinned GPS positions to 24‐h intervals (with a 6‐h tolerance) to regularise sampling and reduce temporal autocorrelation. We then divided the movement trajectory into segments of homogenous movement by comparing the mean and variance of latitude and longitude over time using the default settings in the Segclust2d package (Patin et al. 2020). This method is robust to gaps because missing data has a limited effect on the mean or variance in locations (Patin et al. 2020). We used a minimum segment length (L_min_) of 7 locations, representing a duration of 1 week. Stationarity was then visually assigned for segmented movement phases when variograms in the ctmm package (Fleming et al. 2025) reached a stable asymptote, positions were well‐mixed in time, and segments were minimally 30 days in duration. Assigning stationarity and subsequent methods used all GPS positions without thinning. The longest stationary season‐limited segment for each season was then used to estimate individual seasonal home ranges (Figure 1B). Home ranges (and 95% confidence intervals) were estimated using the optimally weighted and area‐corrected autocorrelated kernel density estimator (wAKDEc) in the ctmm package. The underlying movement models were estimated and selected using the ctmm.select function in the ctmm package, using the perturbative hybrid residual maximum likelihood (pHREML) parameter estimator (Fleming et al. 2025; Silva et al. 2022). Because this method is based on a continuous time movement model, it accounts for gaps and temporal autocorrelation in the data and therefore allows for the comparison of home range estimates based on different sampling durations and intervals. We hereby assume that gaps in GPS positions are random and do not systematically bias, or obscure, fox movement behaviour. We consider this a reasonable assumption given the relatively short maximum gap length during home ranging periods (Table 2) and the fact that gaps were caused by transmission problems in only a subset of collars. Home range estimates with 95% confidence intervals, effective sample sizes and the fitted underlying movement models and variograms are illustrated in Appendix S1.
  Seasonal variation in home range size was tested with a paired *t*‐test for five foxes that stayed in the same area during both seasons by testing for the difference in the natural logarithm of the home range size estimate between summer and winter. We determined seasonal home ranges to be in the same area by quantifying their overlap using the overlap function in the ctmm package and using a minimum Bhattacharyya coefficient value of 0.5.
  To test the combined effects of season and environmental conditions within the home range on home range size, we used linear mixed‐effects models as implemented in the lme4 package (Bates et al. 2025). The response variable was the natural logarithm of the home range size estimate, with individual foxes included as a random effect to account for repeated measures. Season (summer/winter) and one of the three continuous environmental variables were included as fixed effects: (1) LogCoastDist at the home range centroid, (2) mean EVI and (3) mean LUI within the home range extent. Due to the limited sample size, each environmental variable was tested in a separate model to avoid overfitting.
2. Maximum net displacement: This method incorporated all types of movement behaviour and calculated the maximum distance between two GPS positions per individual within a weekly sliding window (requiring at least five sampling days), per month (requiring at least 20 sampling days) and over the entire tracking duration. Maximum net displacement values per week and month were visualised over an annual cycle to describe temporal patterns.

#### 2.4.2 Habitat Selection

We used Resource Selection Functions (RSFs) to analyse habitat selection for our three environmental variables (EVI, LogCoastDist and LUI) within individual home ranges (3rd order selection). RSFs were constructed using the ctmm package (Fleming et al. 2025), incorporating autocorrelation‐informed likelihood weighting of locations to address temporal autocorrelation‐induced pseudoreplication and bias in parameter estimates (Alston et al. 2023). The geographic domain of availability in this method is a Gaussian home range estimate that approximates the maximum likelihood estimate of the autocorrelation model (Alston et al. 2023). For each individual home range, we used the rsf.select function and primarily used models that included all three environmental variables. However, exceptions were made in specific cases: (1) when multicollinearity was detected (Pearson's *r* > 0.7), we selected the best‐supported model that did not include both correlated variables; and (2) when the coastline was beyond the geographic domain of availability, the logarithm of the distance to the coast was excluded from the model.

We further estimated summer and winter population selection parameters using the mean function in ctmm package. This method used a hierarchical model to estimate population‐level parameters from individual‐level RSF models. Modelling the summer and winter populations separately allowed us to capture potential variation in selection patterns between the seasons.

## 3 Results

### 3.1 Home Range Size

**FIGURE 2:**
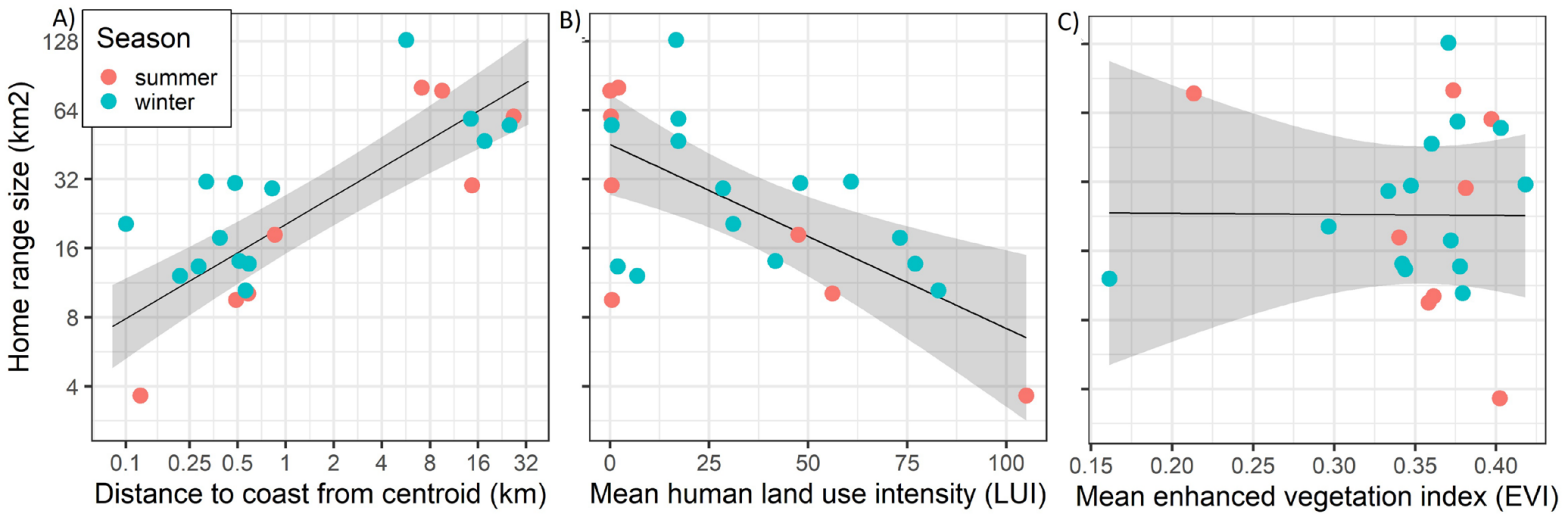
Effects of predictors (A) the natural logarithm of the distance to the coastline (LogCoastDist), (B) human land use intensity (LUI) and (C) Enhanced vegetation index (EVI) on the natural logarithm of the home range size. Grey shaded areas show 95% confidence intervals of the three separate linear mixed‐effect models which used season as additional fixed effect and individual as random effect. The y‐axes, and x‐axis in the first panel have been back transformed from log scale to improve readability.

Estimated seasonal home range sizes averaged 35 km^2^ across both seasons, but variation was substantial; from a minimum of 3.7 km^2^ (95% CI: 3.3–4.0) to a maximum of 129 km^2^ (95% CI: 109.3–150.3). Home ranges were found to be significantly larger in winter compared to summer, as indicated by the paired *t*‐test and the linear mixed‐effects model that included LogDistCoast as an environmental predictor variable and individual as a random effect. Taking the exponent of the coefficient estimates (Table 2) indicates that home ranges were estimated to be 37% (95% CI; 1.5%, 84%) to 67% (95% CI; 20%, 128%) larger in winter than summer, respectively for each statistical test. More uncertainty in the effect of season on home range size was estimated by the linear mixed‐effect models that included LUI or EVI as environmental predictor variables (Table 2). The models also indicated that home ranges were significantly larger farther from the coastline, and significantly smaller at higher mean LUI. Taking the exponent of the coefficient estimates (Table 2) indicates that home ranges were estimated to be 33% larger (95% CI; 24%, 42%) at twice the distance to the coast, and 1.8% smaller (95% CI; 0.9%, 2.8%) for every unit increase in LUI (Figure 2). No effect of EVI on home range size was found (Table 2, Figure 2). Home range size estimates per season, including additional information on individual foxes, are provided in Appendix S2.

### 3.2 Displacement Distance

**FIGURE 3:**
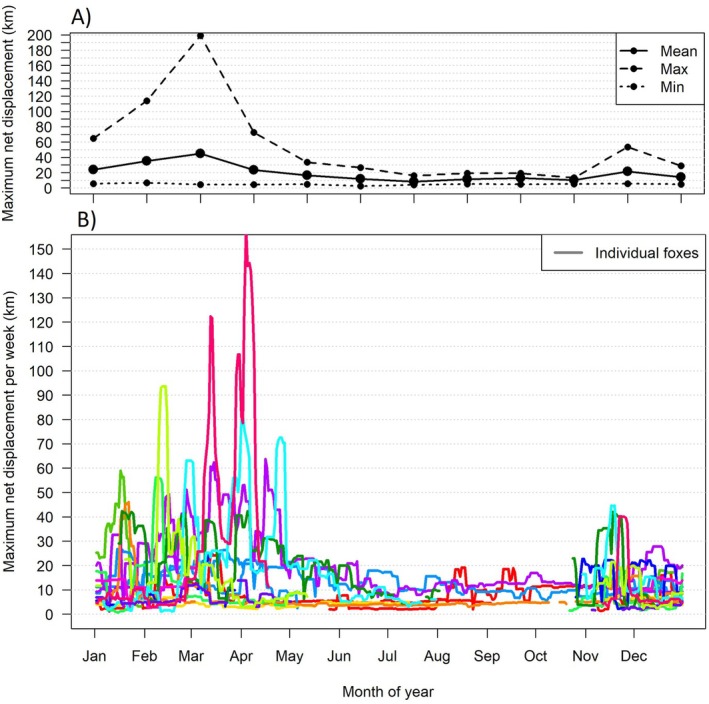
Maximum net displacements over an annual cycle. (A) Per month of the year, summarised over all individuals in mean, maximum and minimum values. (B) Per weekly sliding window, colours represent the same individual foxes as shown in Figure 1.

Maximum net displacement distance on monthly and weekly timescales varied clearly throughout the annual cycle but showed substantial variation between individuals and within individuals over time. Maximum net displacement per month ranged from 2.6–33 km in May–October to 4.5–199 km in November–April (Figure 3A). This variation was best described on weekly timescales: a maximum net displacement below 10 km per week was common in all individuals and during all months of the year, but these periods of relatively limited movement alternated with bouts of movement > 40 km/week in 8 out of 14 individuals (including both sexes and ages) in the period from November–April. From May–October, displacements remained below 25 km per week (Figure 3B). Over the entire tracking duration maximum net displacement varied greatly, from 8 to 337 km (Table 1).

### 3.3 Habitat Selection

**FIGURE 4:**
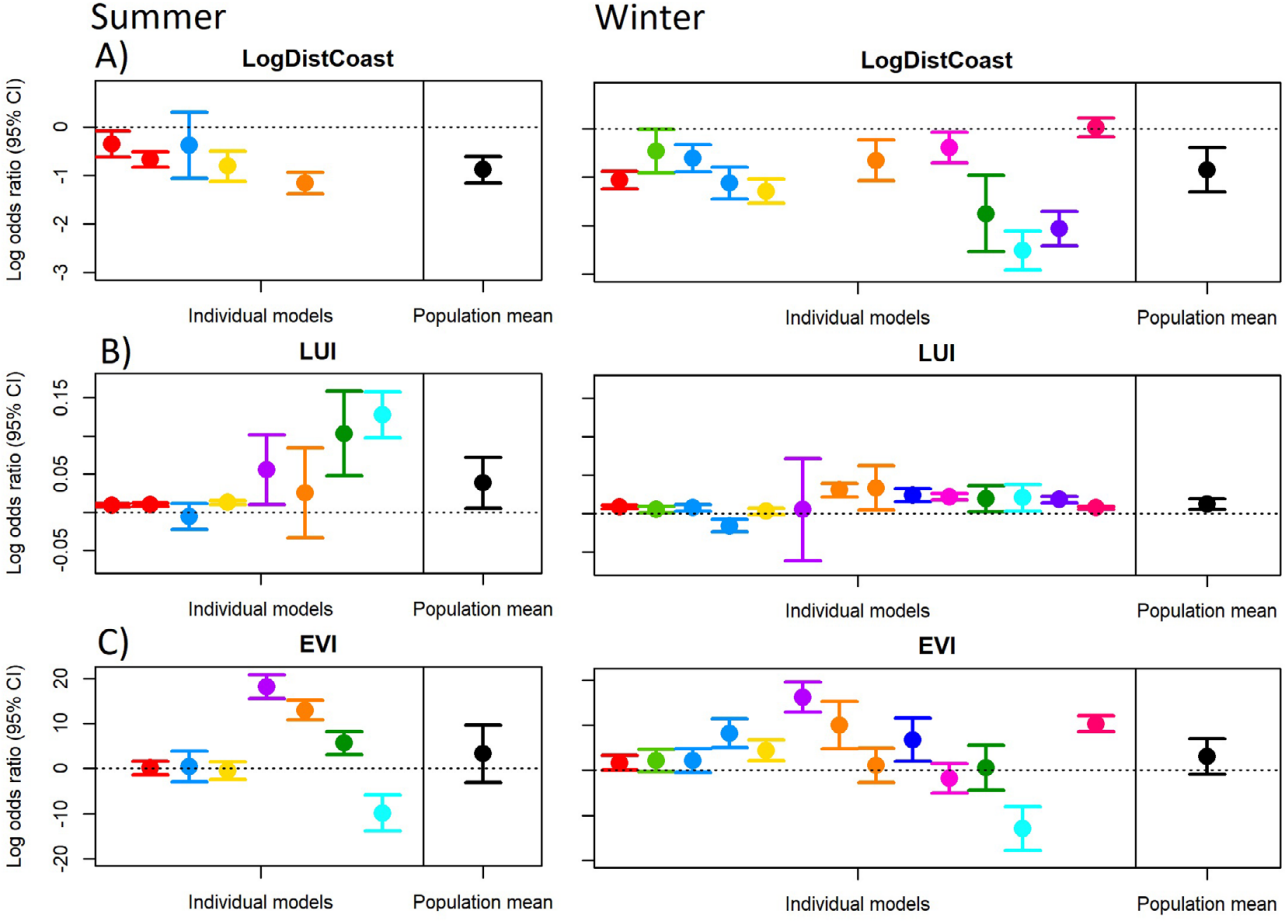
Third order habitat selection model coefficients (in log odds ratios) with 95% confidence for environmental predictors (A) the natural logarithm of the distance to the coastline (LogDistCoast), (B) human land use intensity (LUI) and (C) Enhanced vegetation index (EVI) during both summer and winter seasons. Results are shown for both individual RSF models, and population means for the respective environmental variables and seasons. Colours represent the same individual foxes as shown in Figure 1.

Resource selection functions revealed that foxes at the population level significantly selected for habitats in closer proximity to the coastline and for habitats with higher LUI in both summer and winter home ranges (Figure 4). At twice the distance to the coast, the odds of habitat use at the population level decreased by about 44% in winter (coefficient; log odds ratio for 1 unit of change = −0.848; 95% CI: −1.306, −0.390) and 45% in summer (coefficient = −0.881; 95% CI: −1.154, −0.607). For every unit increase in land‐use intensity, the odds of habitat use at the population level increased by about 1.2% (coefficient = 0.012, 95% CI: 0.006, 0.019) in winter and by 4.0% (coefficient = 0.039, 95% CI: 0.006, 0.072) in summer. At the individual level, most foxes selected for habitats closer to the coast and habitats with higher LUI, but the strength of this selection varied (Figure 4).

The odds of habitat use did not increase significantly with increasing EVI. The effect size showed large individual variation and was not significantly different from zero at the population level (coefficient winter = 3.043, 95% CI: −0.871, 6.958, coefficient summer = 3.289, 95% CI: −3.062, 9.640) (Figure 4).

## 4 Discussion

### 4.1 Geographic Space Use

Expected food resource density, linked to environmental productivity and seasonality, is a well‐known determinant of home range size in carnivores including red foxes (Herfindal et al. 2005; Main et al. 2020; Walton et al. 2018). Our findings support this notion, with seasonal home range sizes averaging 35 km^2^, falling into the upper range of what has been documented worldwide for the red fox (Main et al. 2020). This also aligns with estimates from other tundra regions where food resource densities are expected to be relatively low (Lai et al. 2022; Warret Rodrigues and Roth 2023). However, we found substantial variation in home range sizes throughout the study area: from 3.7 km^2^ up to 129 km^2^. We were able to link this variation to environmental and seasonal factors.

As expected, red fox home ranges were found to be significantly smaller closer to the marine coastline. On the coastline, marine‐derived food items, such as seabirds or carrion, become available to terrestrial predators and can provide important food subsidies, especially in low productive ecosystems like the Arctic tundra (Gauthier et al. 2011). Indeed, marine foods are known to be an important dietary component to red foxes in our study area (Killengreen et al. 2011). Food resource densities were therefore expected to increase towards the coastline, reducing the required home range size as predicted by the resource dispersion hypothesis (Macdonald 1983). These findings align with those from High‐Arctic Svalbard, where increased food resource densities at the coast led to decreased home range size in arctic foxes (Eide et al. 2004). Similarly, we expected and found significantly smaller home ranges at higher mean human land use intensities. Human‐derived food subsidies, such as waste or roadkill, tend to be concentrated in space, reducing the required foraging time and area for facultative scavengers (Oro et al. 2013). Consequently, reduced home range sizes in human‐impacted environments are a well‐described phenomenon among generalist carnivores including red foxes (Bino et al. 2010; Main et al. 2020; Šálek et al. 2015). Contrary to expectations however, we found no effect of mean terrestrial vegetation productivity (EVI) within the home range (expected to increase prey densities) on home range size. Marine and/or human impacts thus potentially override the effects of terrestrial ecosystem productivity on red fox home range size on a local scale, as found by Main et al. (2020) globally. Alternatively, while variation in EVI values was substantial at small spatial scales within our study area, mean EVI values at the home range scale potentially varied insufficiently to capture meaningful differences in prey densities. Furthermore, while remotely sensed vegetation productivity variables are commonly used as a proxy for resource density in carnivore studies (Herfindal et al. 2005), not all food resources consistently correlate with vegetation productivity, as is the case for reindeer carcasses, lemmings and grey‐sided voles in our study area (Soininen et al. 2018).

In response to temporal variation in resource availability, animals may either expand their home ranges (flexible strategy) or maintain stable home ranges large enough to meet minimal needs in times of food scarcity (obstinate strategy) (Von Schantz 1984). Our results suggest that red foxes on Varanger used a flexible strategy, with home ranges estimated to be 37% to 67% larger in winter compared to summer. These results are in line with findings from the Canadian Arctic tundra where red fox home ranges were estimated to be 90% larger in winter (Warret Rodrigues and Roth 2023). While red foxes are territorial animals, territorial defence is thought to diminish with increasing area of the home range (Goszczyński 2002). The potential for flexible space use strategies may thus be higher in areas with larger home ranges and lower densities because increased space use would less likely induce negative territorial interactions. Other social factors such as age, breeding status or sex may further help explain described differences in home range size and seasonal variation therein both within this study and between other studies (e.g., Lai et al. 2022; Meia and Weber 1995; Warret Rodrigues and Roth 2023). For instance, breeding pairs and females in red foxes and other carnivore species have been described to constrict their range during pup rearing, while males may expand their range during the breeding season (Conner et al. 1992; Travaini et al. 1993; Dahle and Swenson 2003). Though investigating these factors was not possible due to limited sample size, a relatively high proportion of subadults in our winter sample may help explain extensive space use during the winter. This is because subadults may not yet hold stable territories and are more likely to disperse (Storm et al. 1976). Maximum net displacement distances further highlighted high mobility during winter. From November to May, approximately half of the tracked foxes (8 out of 14) exhibited occasional movement rates of 40–158 km per week (Figure 3). These foxes moved beyond home range boundaries and either dispersed to new areas, moved nomadically, or conducted round‐trip excursions. As noted by previous authors, extensive winter movement by red foxes in tundra ecosystems may be related to maladaptation of this species to endure periods of food scarcity, forcing long‐distance movement strategies (Warret Rodrigues and Roth 2023). Besides dispersing or moving nomadically in search of better conditions (Warret Rodrigues and Roth 2023), previous research indicates that round‐trip excursions may serve to access shared feeding areas (Carter et al. 2012; Goldyn et al. 2003; Tsukada 1997), mates or new territories (Soulsbury et al. 2011).

### 4.2 Habitat Selection

Consistent with our findings that home range size decreased with increased land use intensity and coastal proximity, foxes were also found to select for these conditions within both their summer and winter home ranges. These results reinforce the notion that human and coastal habitats are important determinants of red fox space use within the tundra. Previous research in similar ecosystems supports these findings. For instance, third order habitat selection of Rocky Mountain red foxes in the US was driven by distance to human features in both summer and winter (Burkholder, Stephenson, Hegg, Gustine, and Holbrook 2025), density of human features being a strong predictor of human‐derived food consumption (Burkholder, Stephenson, Hegg, Gustine, Robinson, and Holbrook 2025). Little is known previously about third order selection for marine coastlines in tundra ecosystems, but this behaviour is well described in red foxes elsewhere (Kimber et al. 2020; O'Connor et al. 2021; Schwemmer et al. 2021). Red foxes typically patrol the tideline for carrion (Schwemmer et al. 2021), a behaviour that was also observed in our study population. Red foxes have been shown to remove over half of the available coastal carrion per night (Brown et al. 2015), underscoring their ability to function as effective coastline scavengers.

Selection for vegetation productivity was uncertain at the population level in both summer and winter owing to large individual variation in this selection. This was surprising because red foxes are often associated with productive landscapes within the tundra (Killengreen et al. 2007; Stoessel et al. 2019) and are known to select for productive shrubland or forest habitat patches within the tundra and elsewhere (Burkholder, Stephenson, Hegg, Gustine, and Holbrook 2025; Cagnacci et al. 2004; Jones and Theberge 1982; Van Etten et al. 2007). Differentiating between distinct vegetation types such as willow thickets and meadows, rather than using a continuous vegetation productivity scale, might better describe how foxes select among variation in tundra vegetation. Furthermore, uncertainty in the selection for vegetation productivity may be explained by the fact that tundra ecosystems are characterised by high temporal and spatial unpredictability in the availability of natural prey, as has been shown for small rodents in the study area (Soininen et al. 2018). Red foxes, being less adapted to these conditions, may therefore more consistently rely on spatially and temporally predictable food subsidies from human or marine sources. Besides seasonal variation, reindeer winter mortality (carrion abundance) and population cycles of small rodents drive yearly variation in terrestrial resource abundance to foxes and this variation was not considered due to the relatively short duration of this study and limited sample size. No obvious variation in movement patterns was observed between those foxes tracked during the peak phase of the rodent cycle (winter 2022–23, *n* = 3), and those tracked during years of lower rodent abundance.

While evidence from previous studies suggests that selection for human, coastal and productive‐vegetation habitats is related to food resource availability, it is important to consider that these habitats may also be utilised for other purposes. For instance, tall vegetation is known to be used for shelter in open landscapes (Black et al. 2025; Lucherini et al. 1995; White et al. 2006), as are human infrastructure (Burkholder, Stephenson, Hegg, Gustine, and Holbrook 2025; Kobryn et al. 2023) and rocky outcrops (Murdoch et al. 2016) which are found along the coastline in our study area. Future studies would do well to consider different behavioural states in habitat selection analyses by using activity sensors or comparing nocturnal to diurnal movement (Black et al. 2025). Finally, consistent selection for human and marine habitats could potentially be associated with sampling bias to individuals predisposed to scavenging due to our use of baited traps.

### 4.3 Management Implications

Red foxes are generalist and opportunistic carnivores that are found in a wide variety of human and natural habitats ranging from semi‐arid deserts across the temperate and boreal zones into the Arctic tundra (Larivière and Pasitschniak‐Arts 1996). Given this broad range, a regionally specific understanding of red fox space use is essential to inform and improve population management strategies (Kobryn et al. 2023). On the Arctic tundra, red foxes were found to be especially mobile in winter, maintaining relatively large home ranges and occasionally displacing beyond home range boundaries. This high mobility suggests that there is little, distance‐wise, to prevent red foxes from moving throughout our study area. Red fox culling, currently conducted in winter along the inhabited coastline, therefore likely affects foxes in the remote inner parts of the peninsula as well. However, high mobility and evidence that immigration is a key contributor to red fox population dynamics in our study area (Nater et al. 2025) suggest that culling should be conducted at even larger spatial scales beyond the size of our study area to achieve long‐term reductions in population size. In contrast, more localised and temporary effects of culling may be achieved if conducted in late spring and summer, when foxes are less mobile. As such, culling efforts could be effective within a target area during a short period of conservation concern (Kämmerle and Storch 2019), such as the breeding season of arctic foxes and ground‐nesting birds. However, since this period coincides with the red fox breeding season, it currently falls outside of the hunting season in our study area. While social factors such as breeding status or sex were not considered in this study, these may be important from a management perspective. For instance, partner removal led to increased space use in raccoon dogs (Toivonen et al. 2025), an unintended side effect in the management of an expanding invader.

Beyond culling, identifying habitat features that are consistently associated with the occurrence of invasive species is critical for achieving targeted and cost‐effective management (Kimber et al. 2020; White et al. 2006). On the Arctic tundra, red foxes are consistently associated with human habitat features across all scales of habitat selection: from their geographic range (Gallant et al. 2020), to the landscape scale (Henden et al. 2014; Rød‐Eriksen et al. 2020), to habitat patches within home ranges (this study), to consumption of human‐derived food items (Savory et al. 2014). Rettie and Messier (2000) proposed that habitat selection reflects an organism's attempt to overcome limiting factors, with the most critical limitations driving selection at progressively finer scales until they are resolved. The persistent selection for human habitats across scales provides strong evidence that human food subsidies play a key role in alleviating the resource scarcity that otherwise limits red fox populations from expanding into low productive tundra ecosystems. Management strategies aimed at reducing the availability of human food resources may therefore provide an effective means of controlling red fox populations in the tundra, as was found elsewhere (Bino et al. 2010). In our study area, foxes may derive human foods from waste, roadkill, slaughter remains or intentional feeding. Understanding which specific types of human‐derived foods are important should be the next step for informing management measures that aim to reduce availability, possibly including improved waste management, public information campaigns or infrastructure modifications. These measures may complement culling efforts and address the underlying drivers of red fox expansion, offering a more sustainable solution to conserving tundra specialists like arctic foxes and ground‐nesting birds.

## Author Contributions

**Stijn P. Hofhuis:** conceptualization (equal), data curation (lead), formal analysis (lead), investigation (equal), visualization (lead), writing – original draft (lead), writing – review and editing (lead). **Arnaud Tarroux:** conceptualization (equal), investigation (equal), supervision (equal), writing – review and editing (equal). **Rolf A. Ims:** conceptualization (equal), funding acquisition (equal), supervision (equal), writing – review and editing (equal). **Dorothee Ehrich:** conceptualization (equal), funding acquisition (equal), investigation (equal), project administration (lead), supervision (lead), writing – review and editing (equal).

## Conflicts of Interest

The authors declare no conflicts of interest.

## Supporting information


**Appendix S1:** ece372512‐sup‐0001‐AppendixS1.docx.

**Appendix S2:** ece372512‐sup‐0002‐AppendixS2.docx.

## Data Availability

GPS positions are available on Movebank (Movebank ID: 5917227474, study name: ‘red fox—COAT—Varanger—GPS telemetry’). All code and additional data sources are archived on OSF, available at https://osf.io/r3hdt/.
