# Supplementary material for: Space Use of an Expanding Generalist Predator Is Shaped by Human, Marine and Seasonal Effects on Arctic Tundra
Source: Ecol Evol. 2025 Nov 17;15(11):e72512. doi: 10.1002/ece3.72512 (PMC12620848; doi:10.1002/ece3.72512)

Appendix 1

The below figures illustrate the home range estimates with 95% confidence intervals, and the variograms of fitted underlying movement models including home range crossing time estimates and effective sample sizes. Figure titles show the respective fox individual, followed by the movement segment number seen in Figure 1, the season, and the underlying movement model (OU =Ornstein-Uhlenbeck, OUF = Ornstein-Uhlenbeck Foraging).


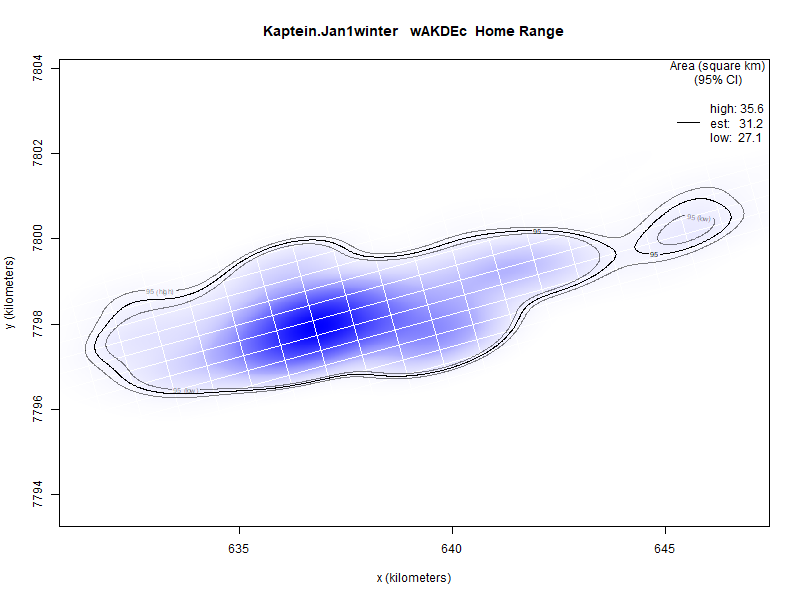

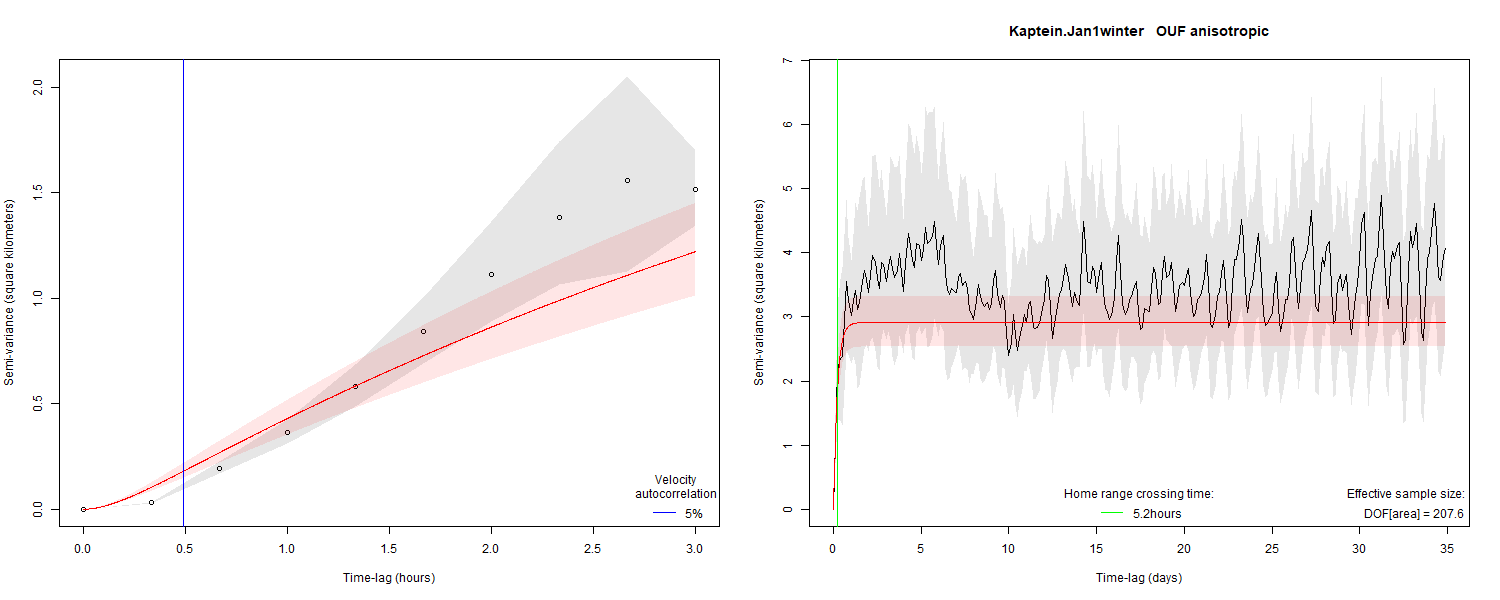

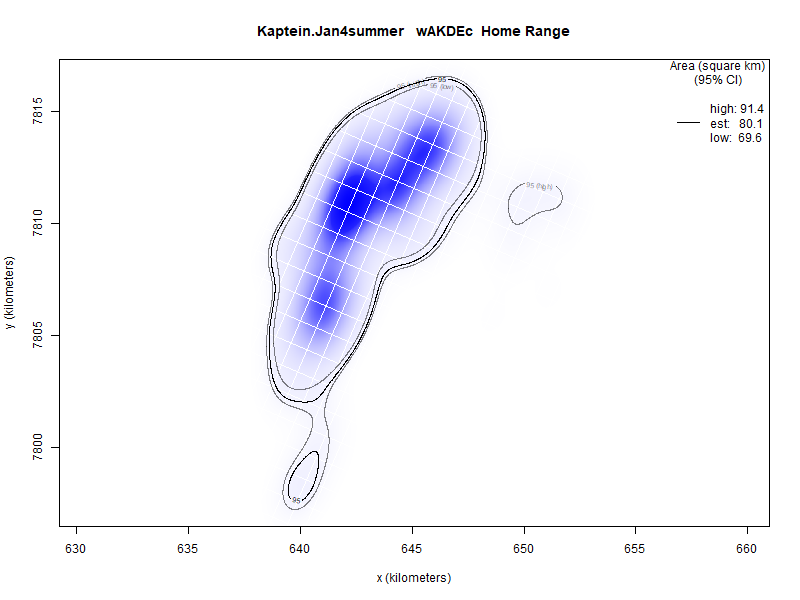

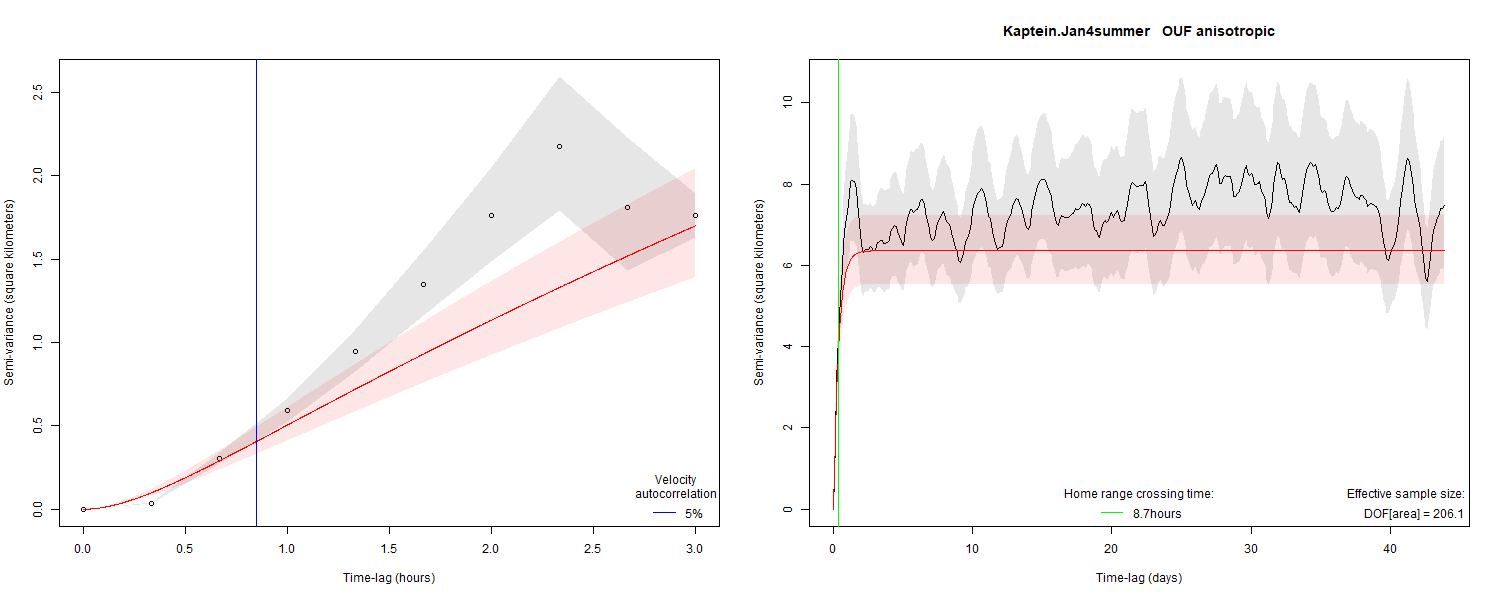

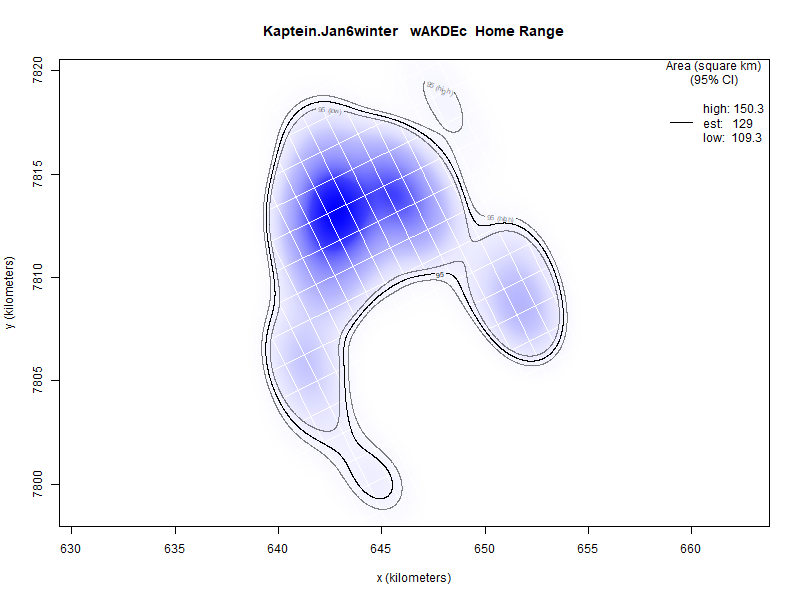

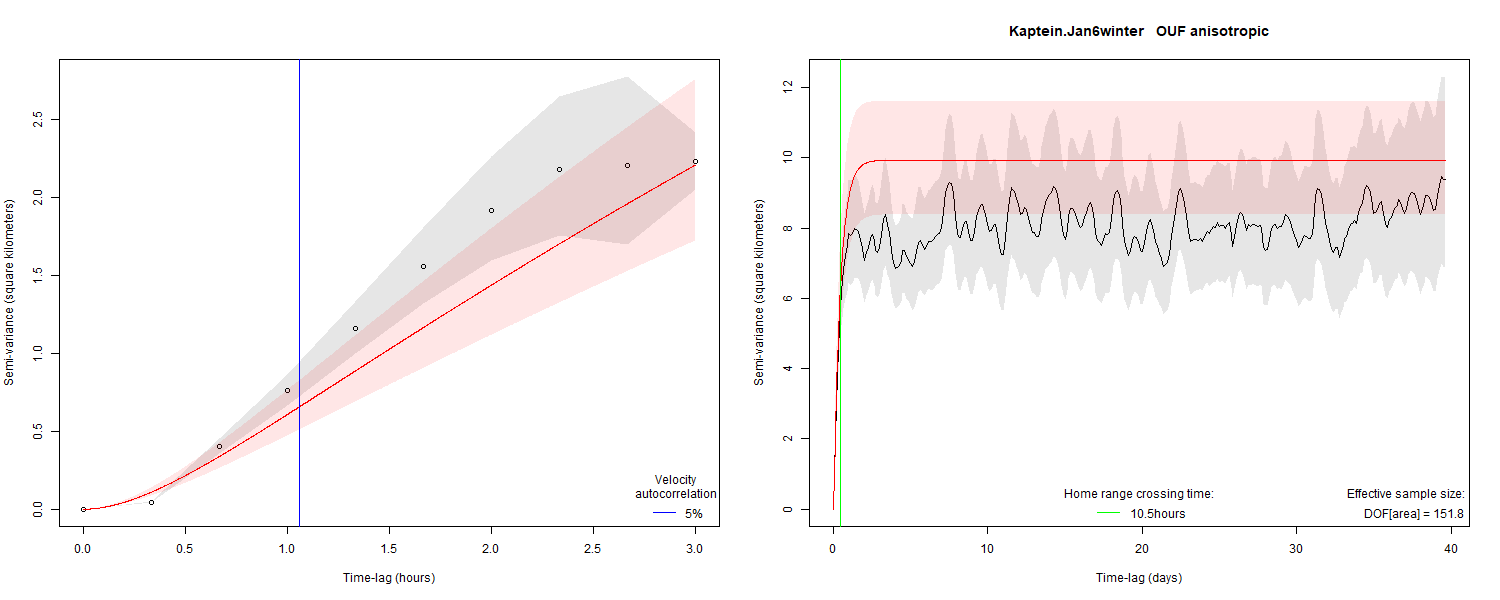

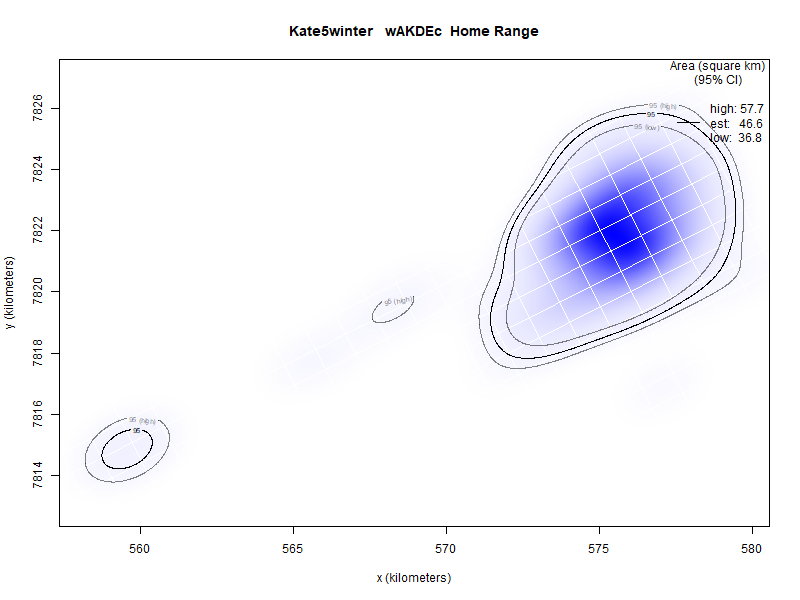

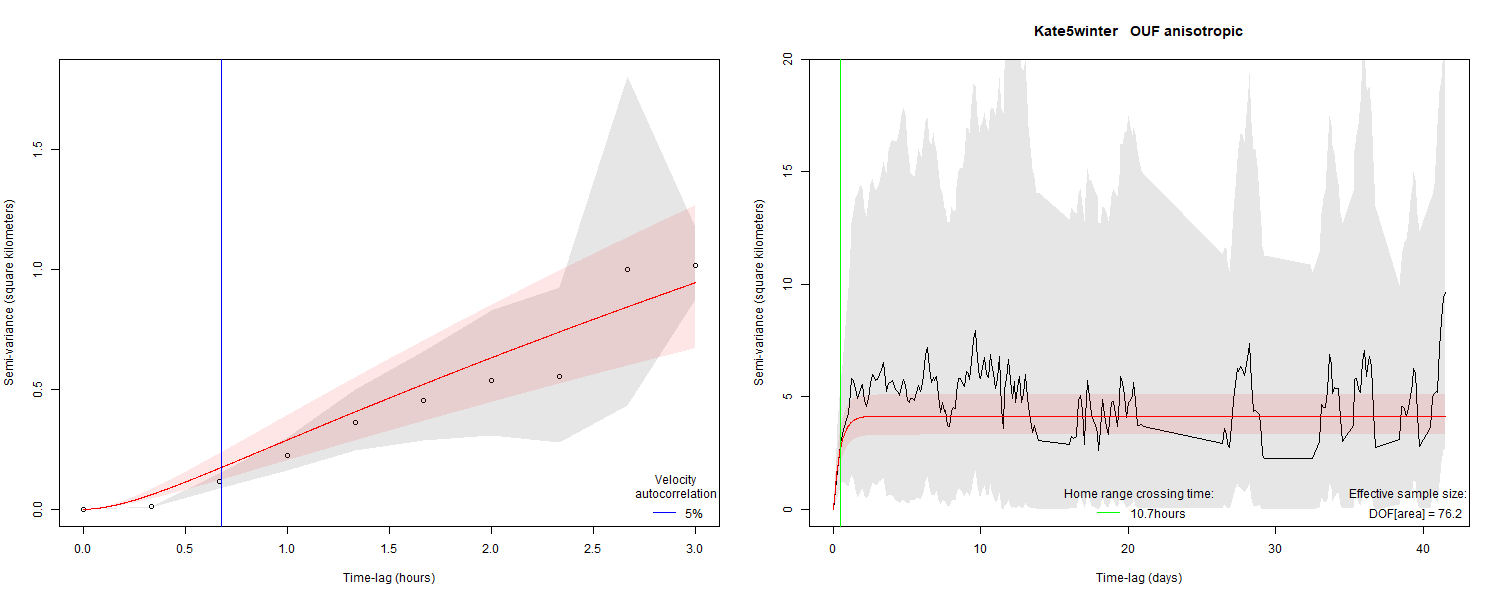

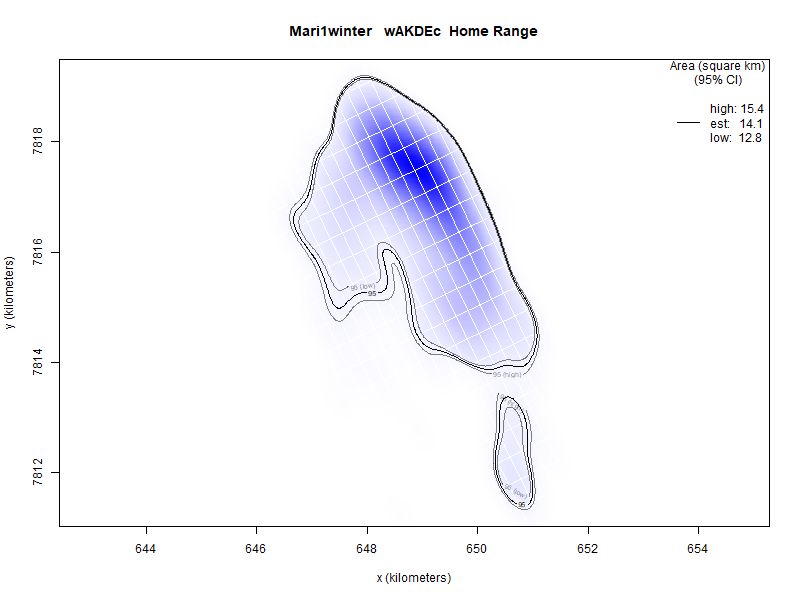

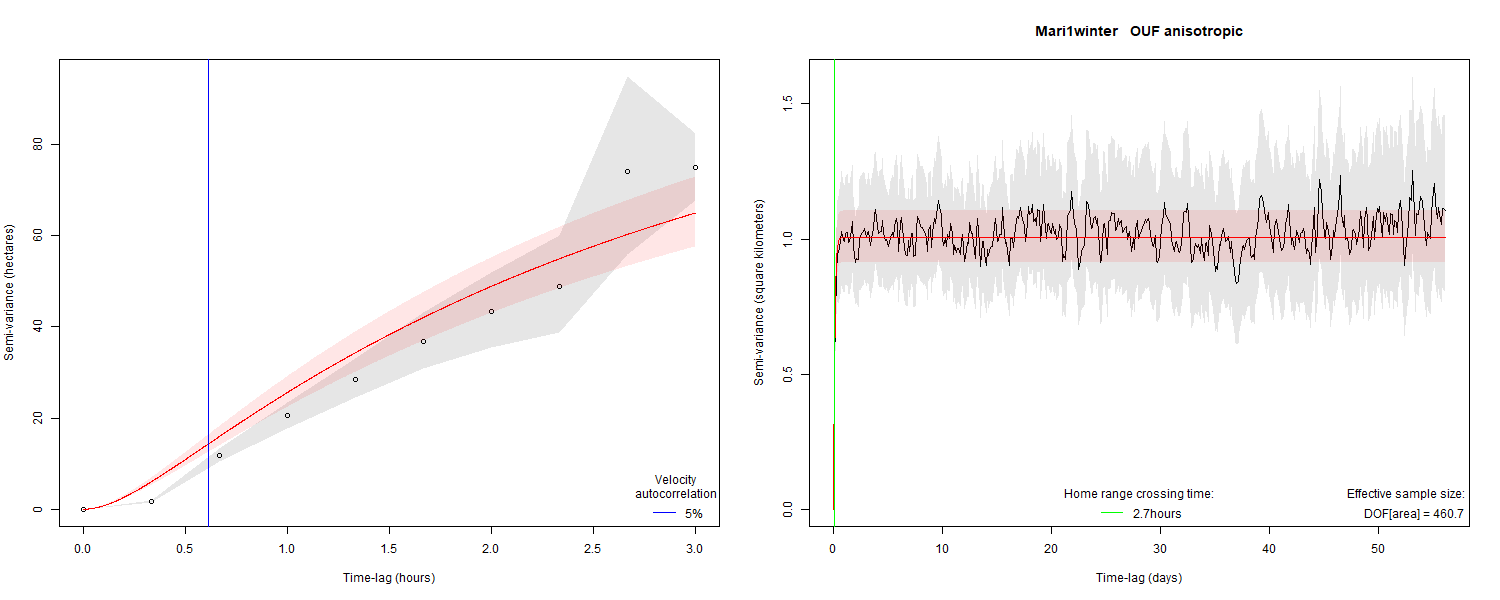

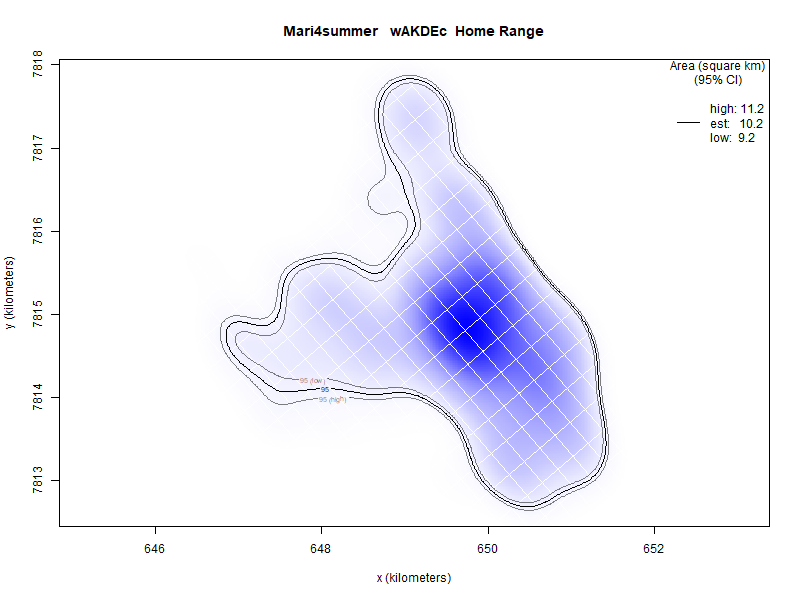

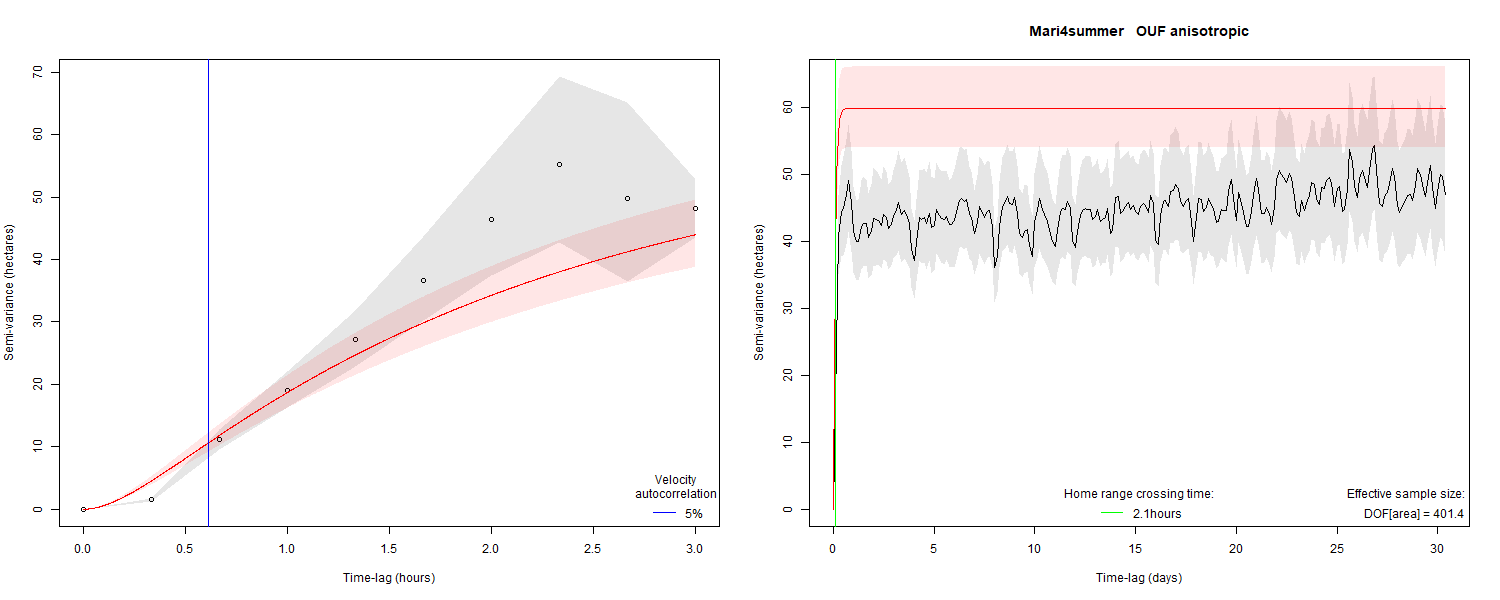

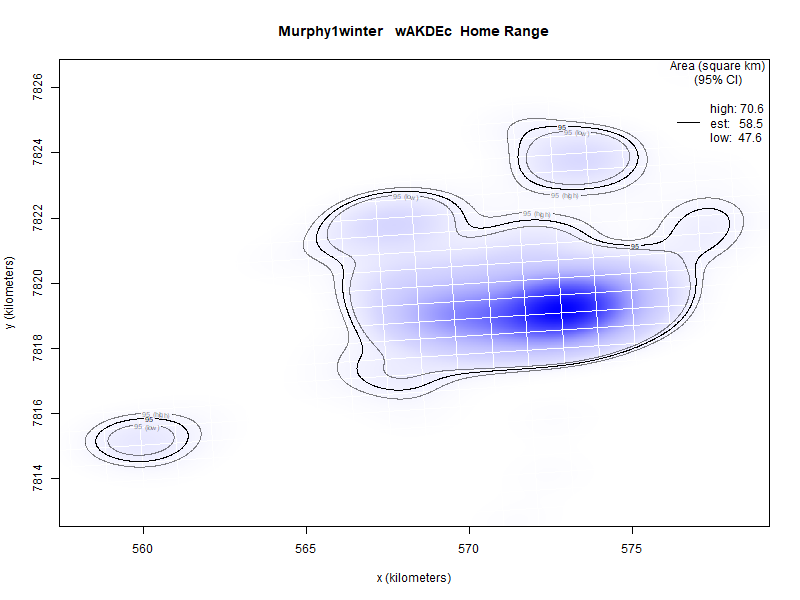

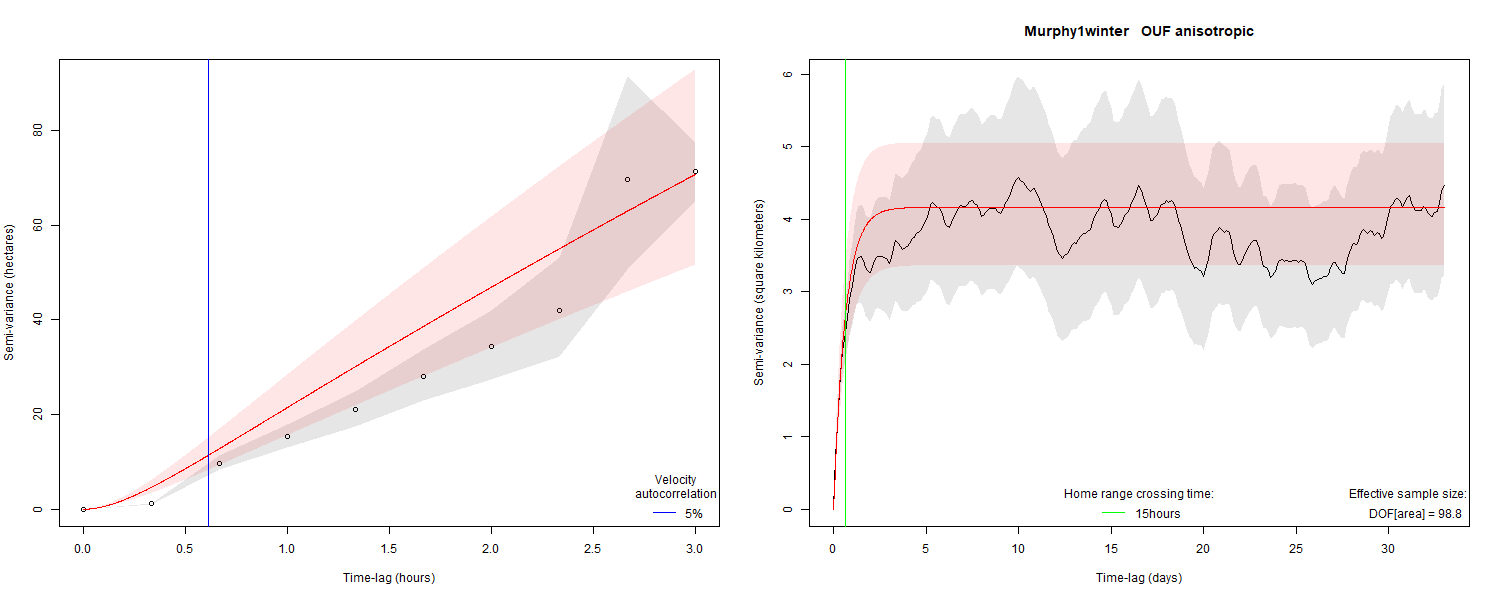

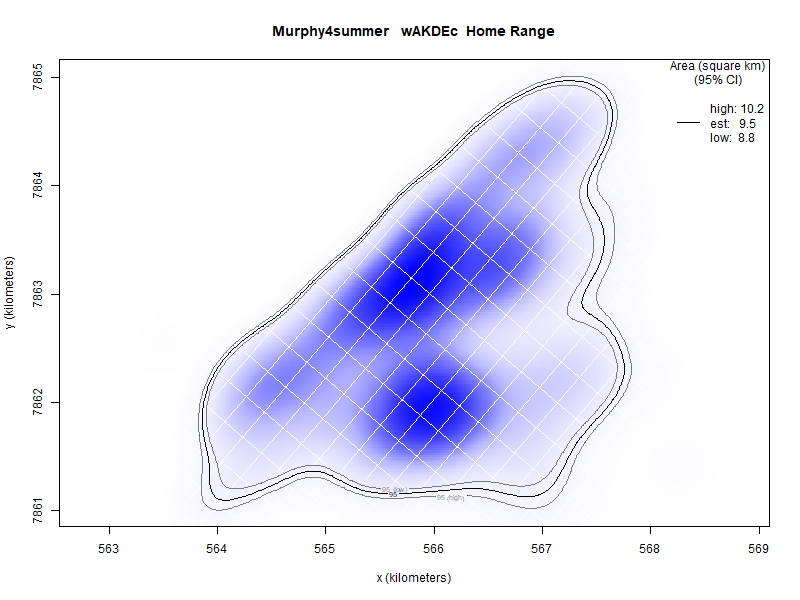

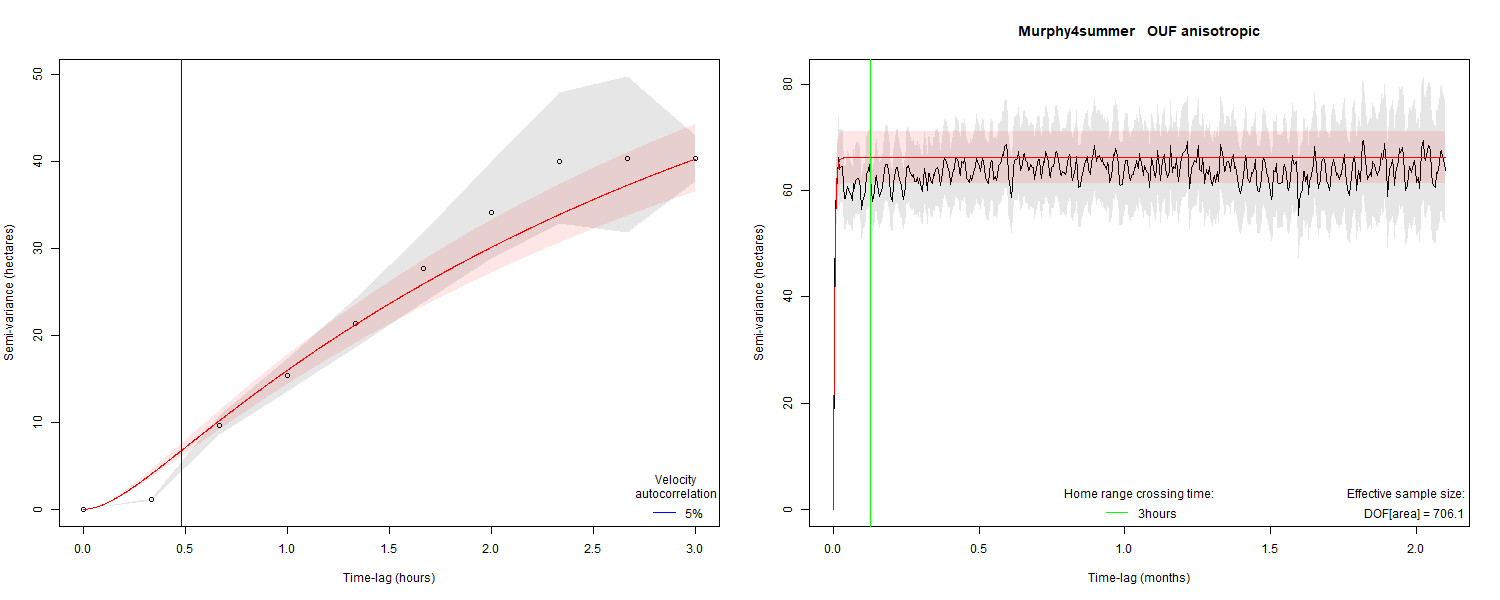

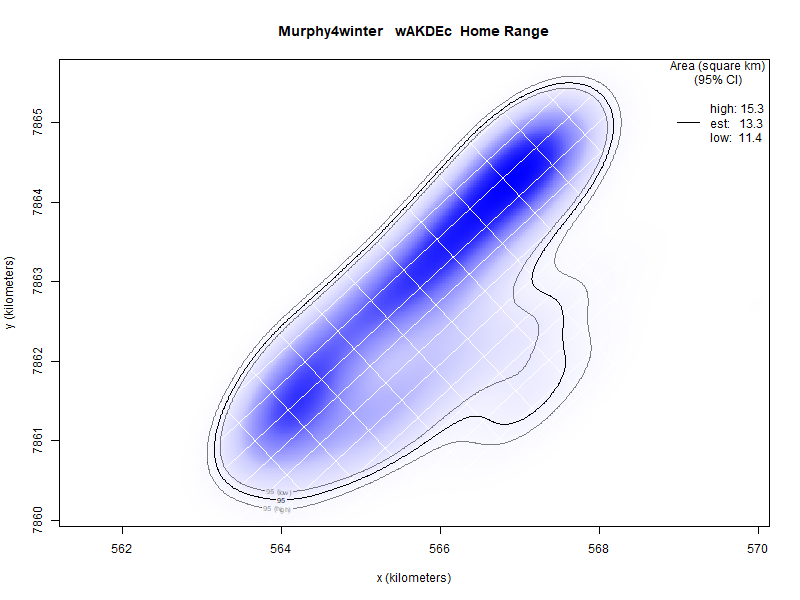

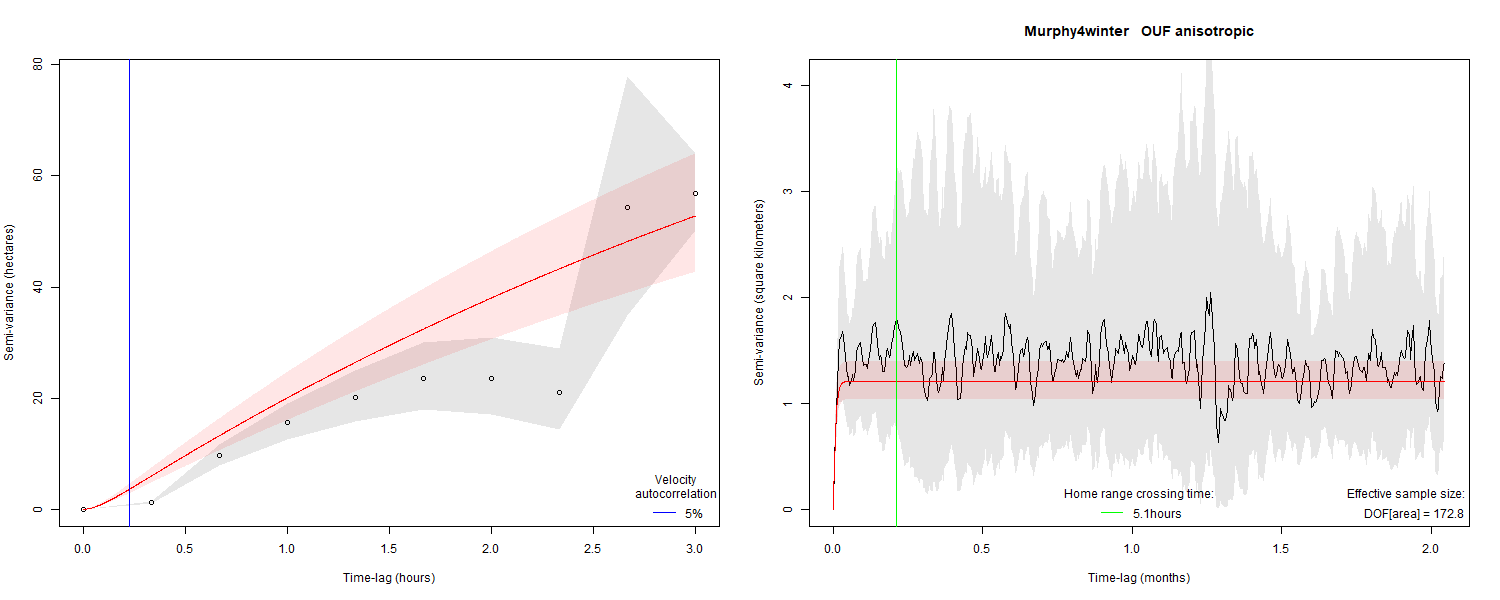

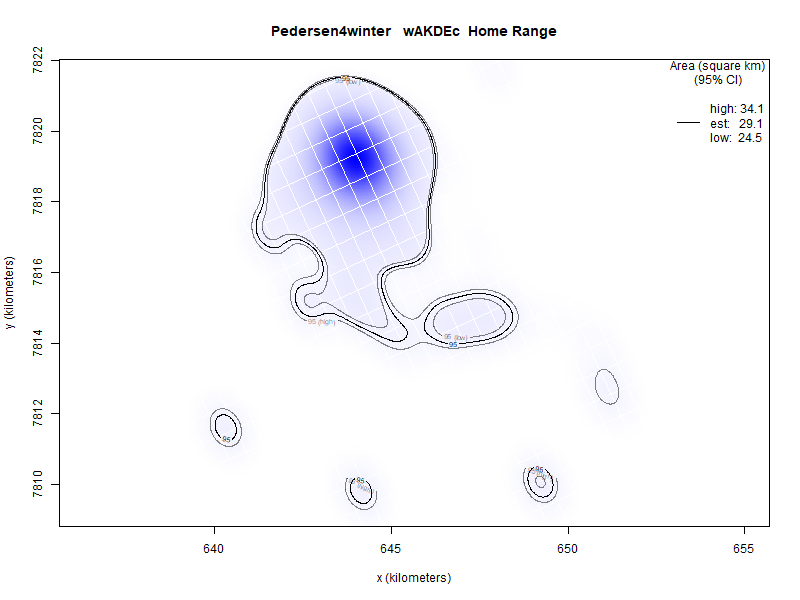

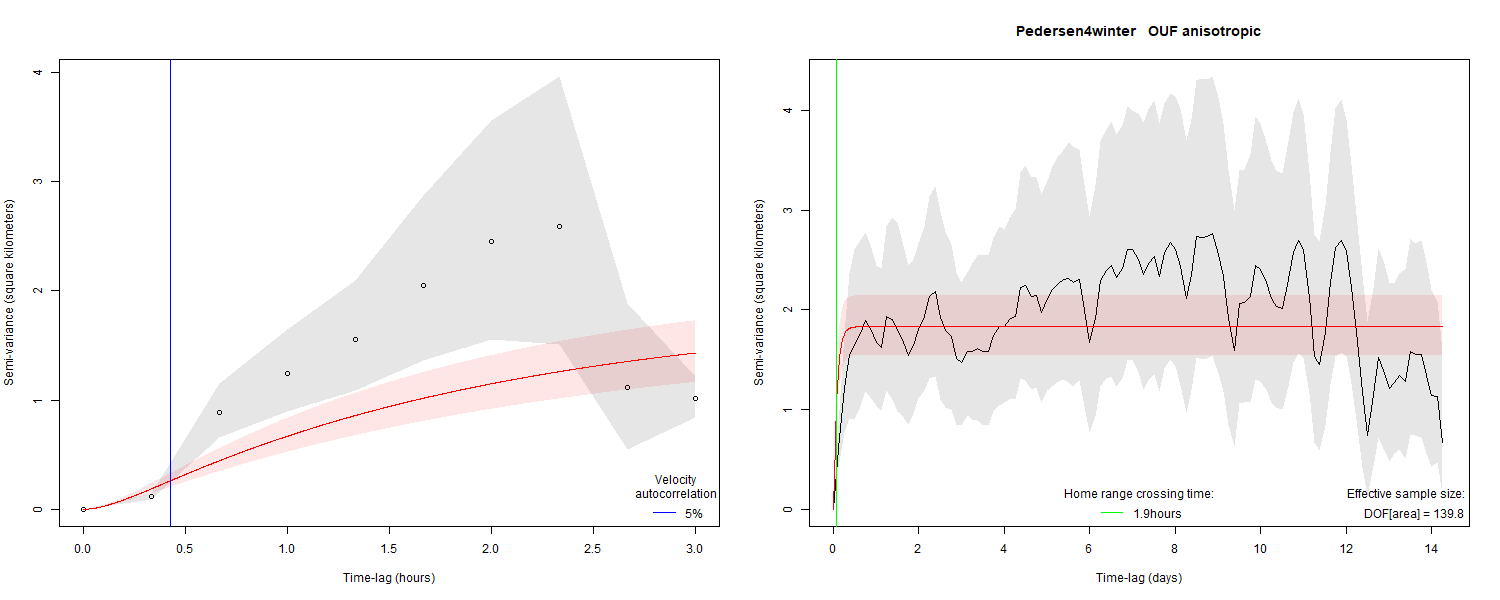

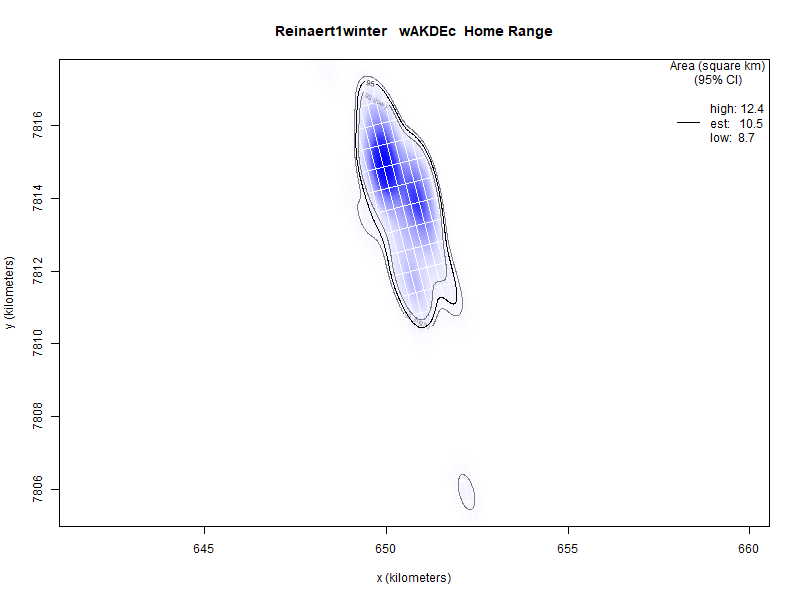

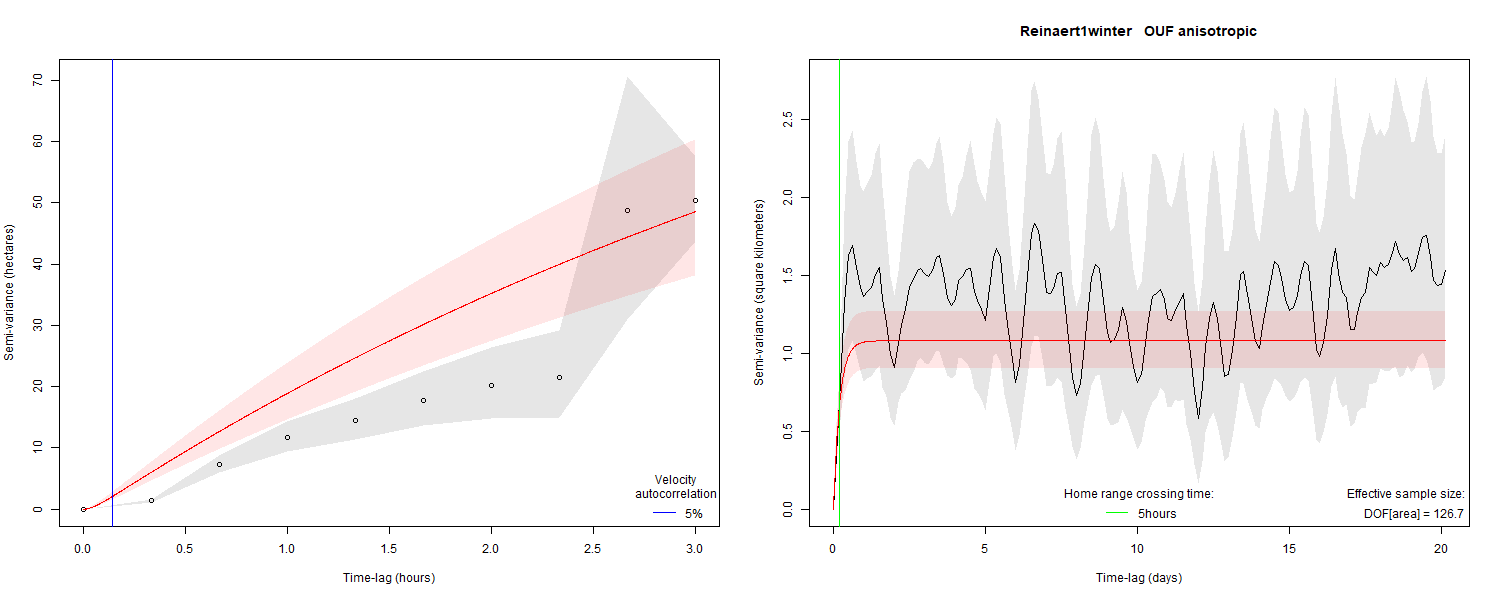

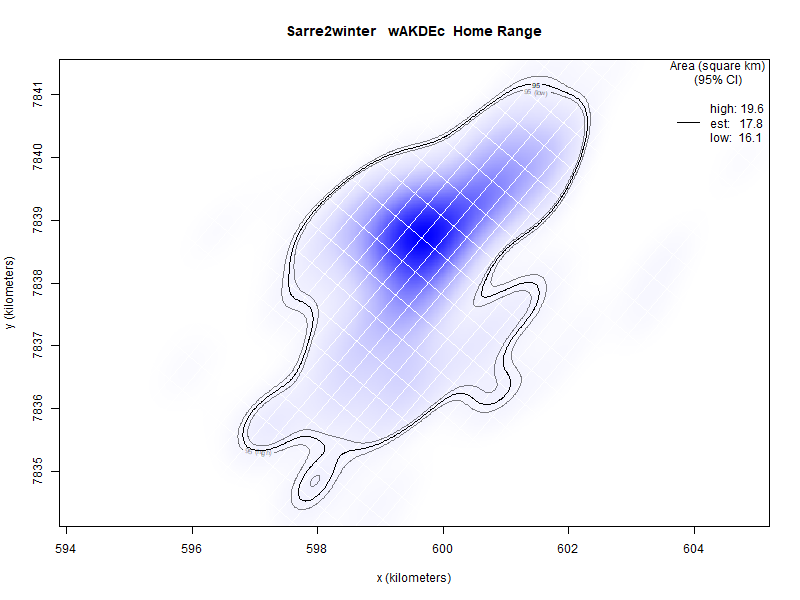

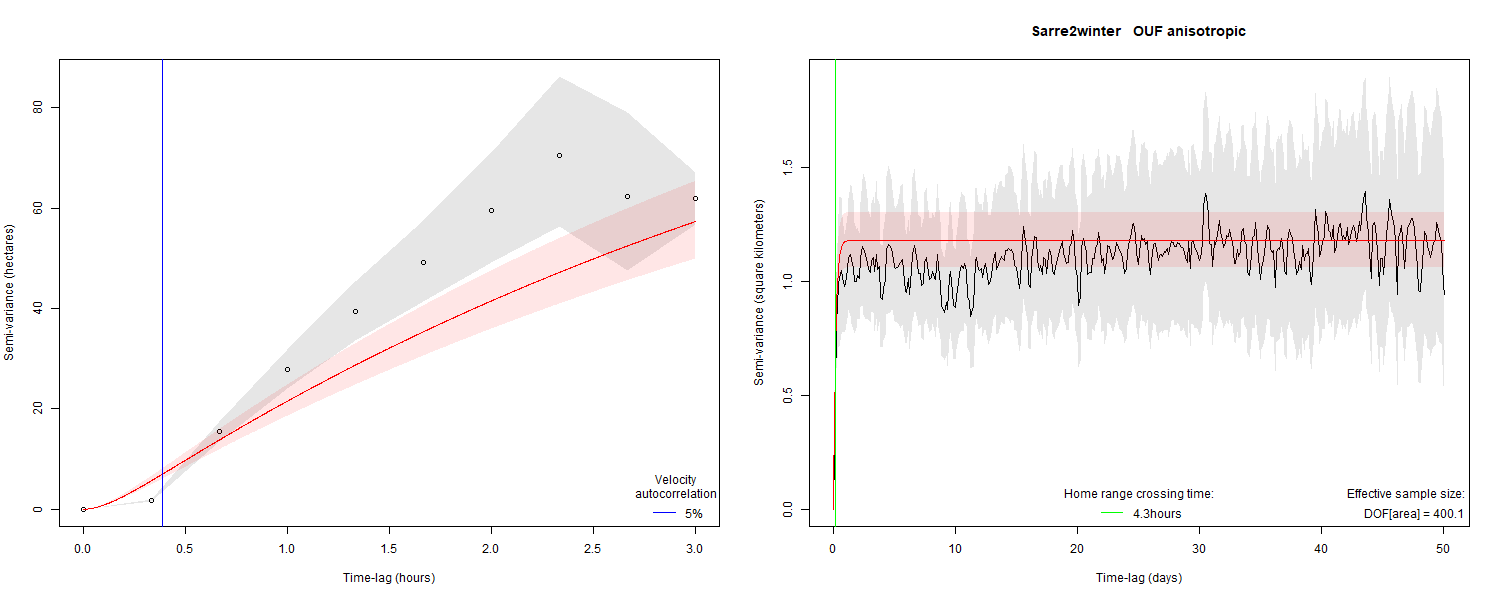

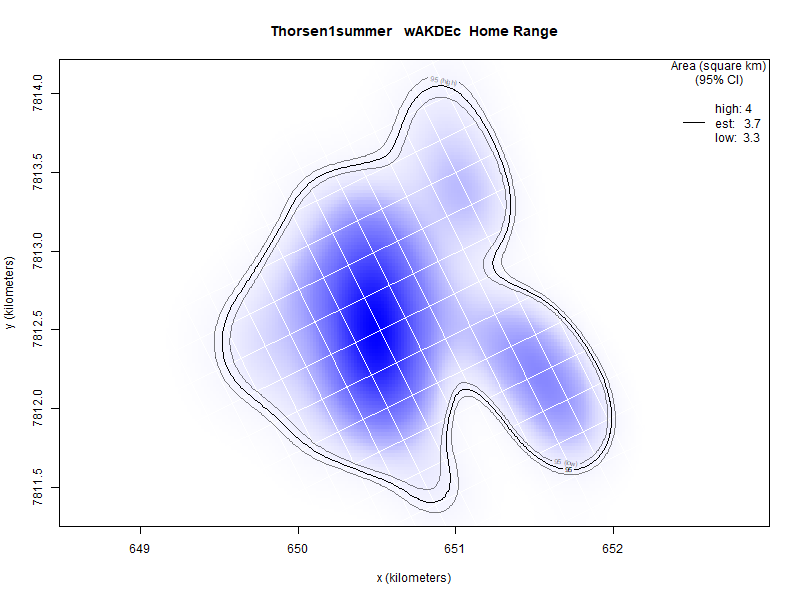

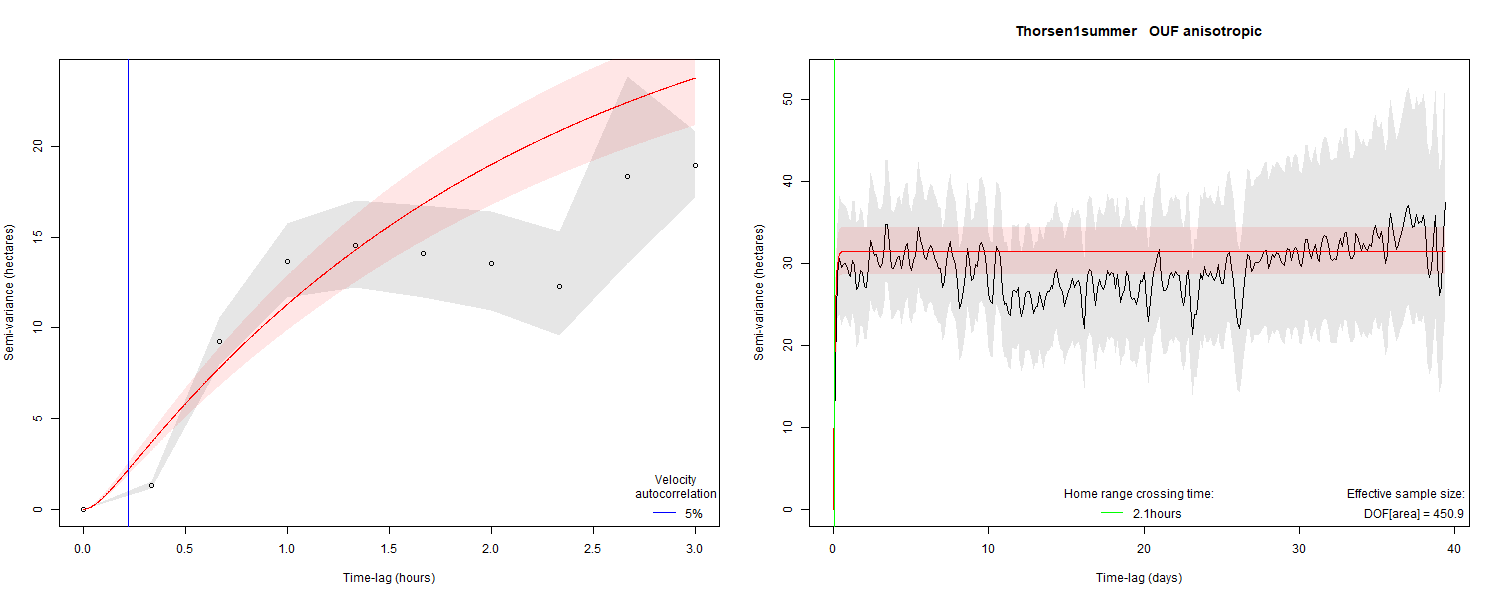

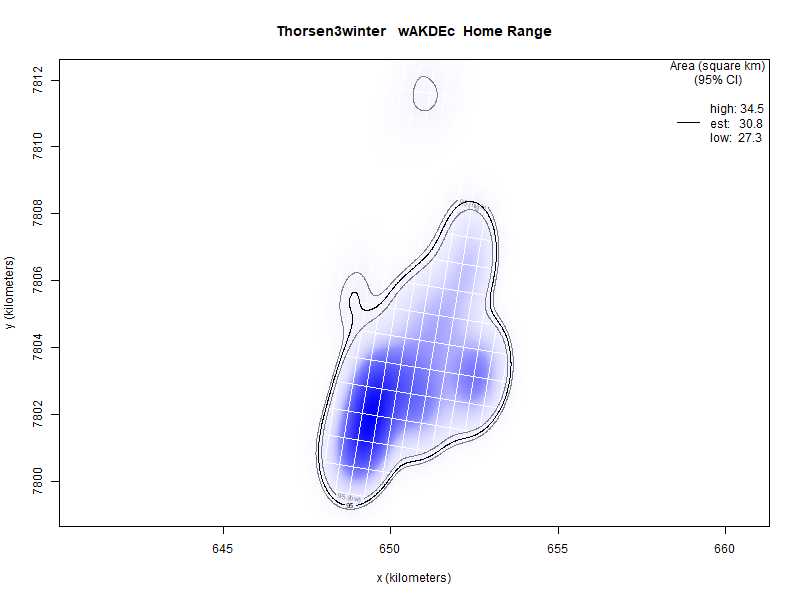

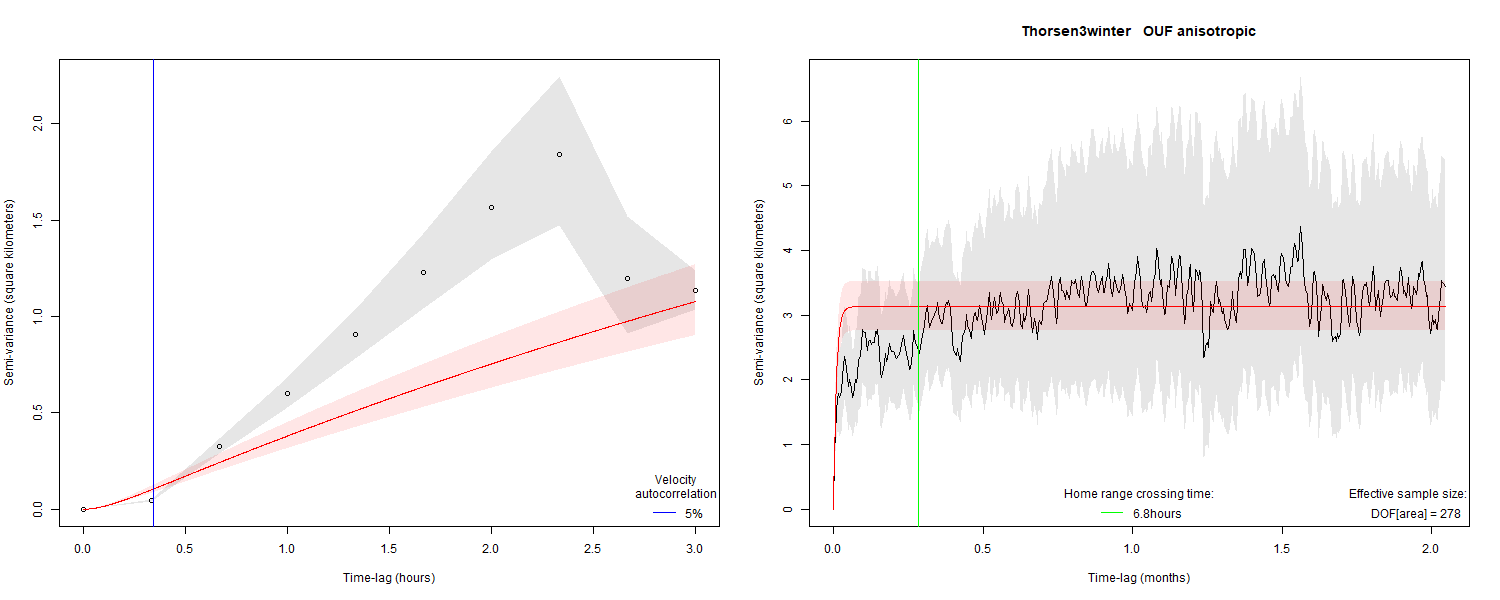

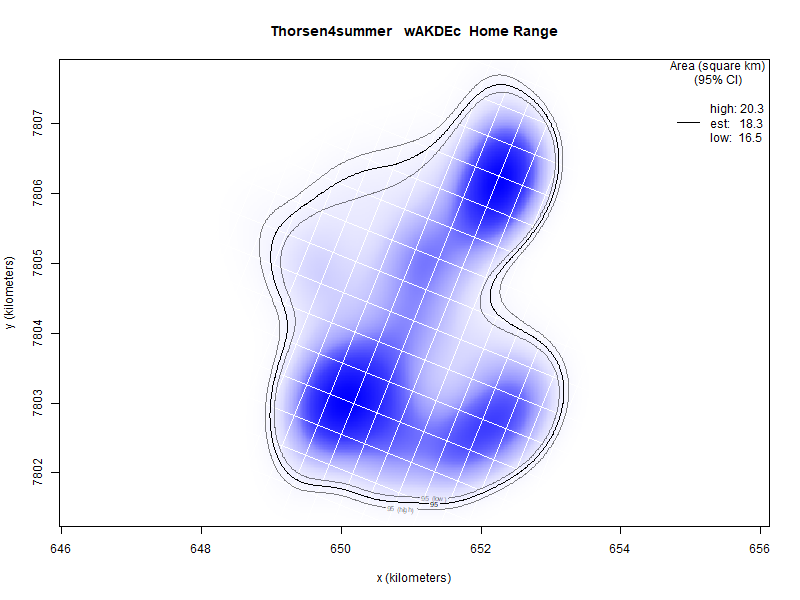

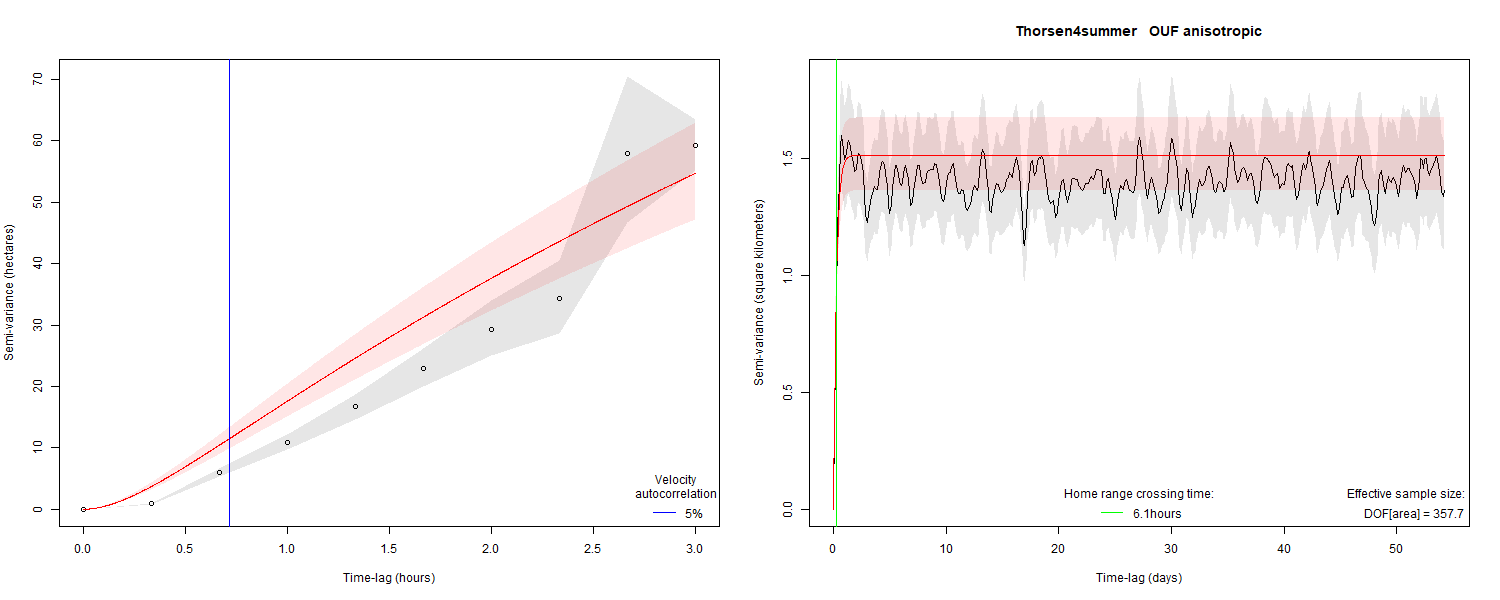

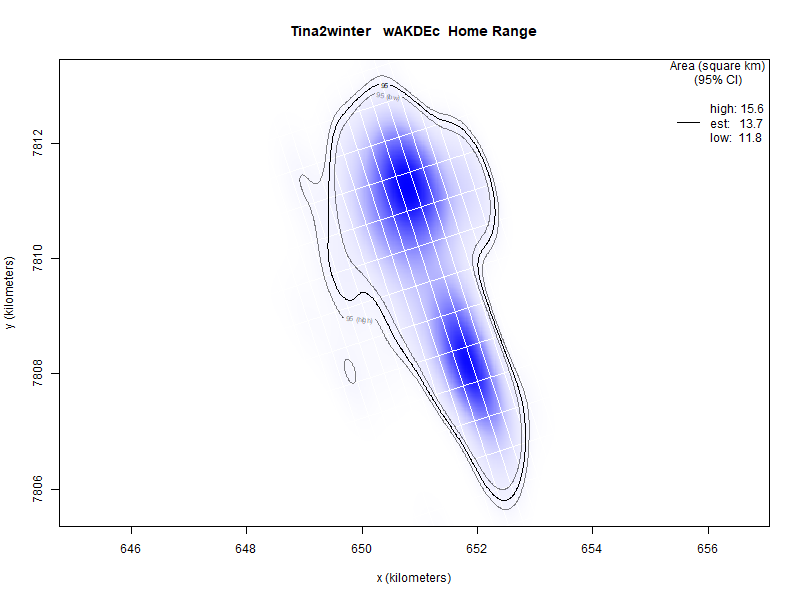

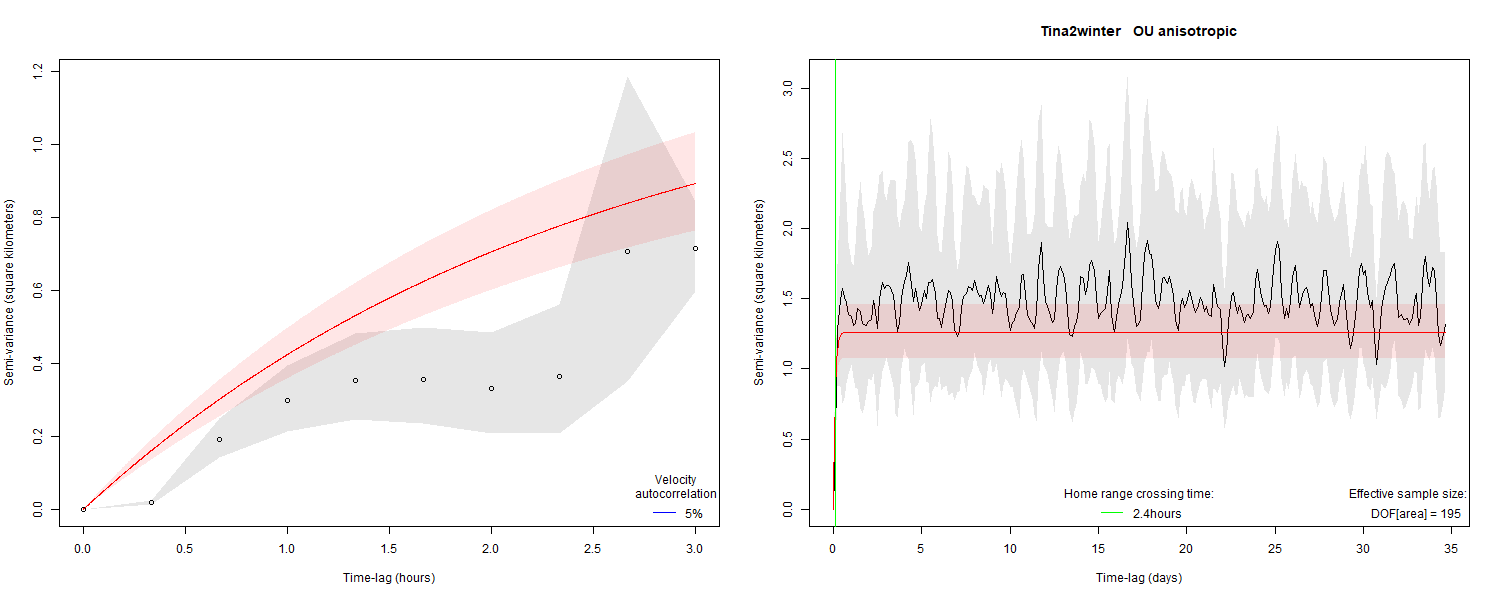

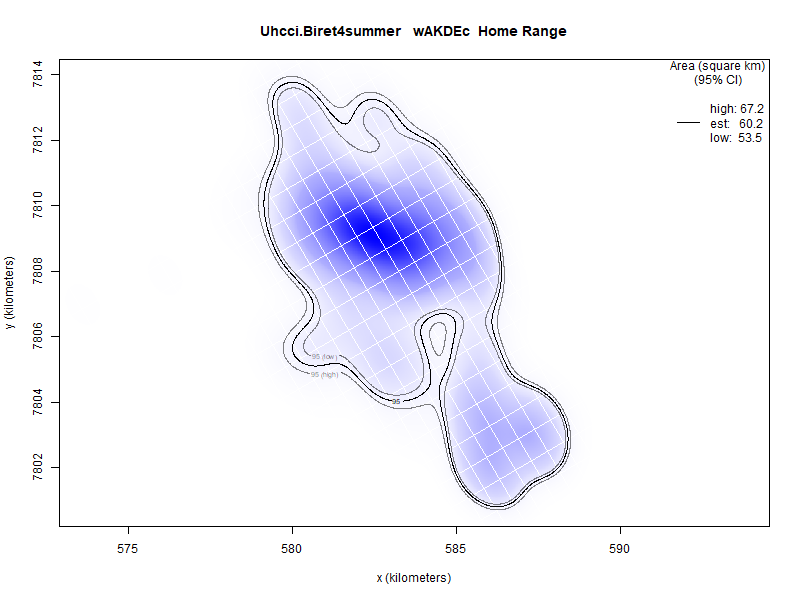

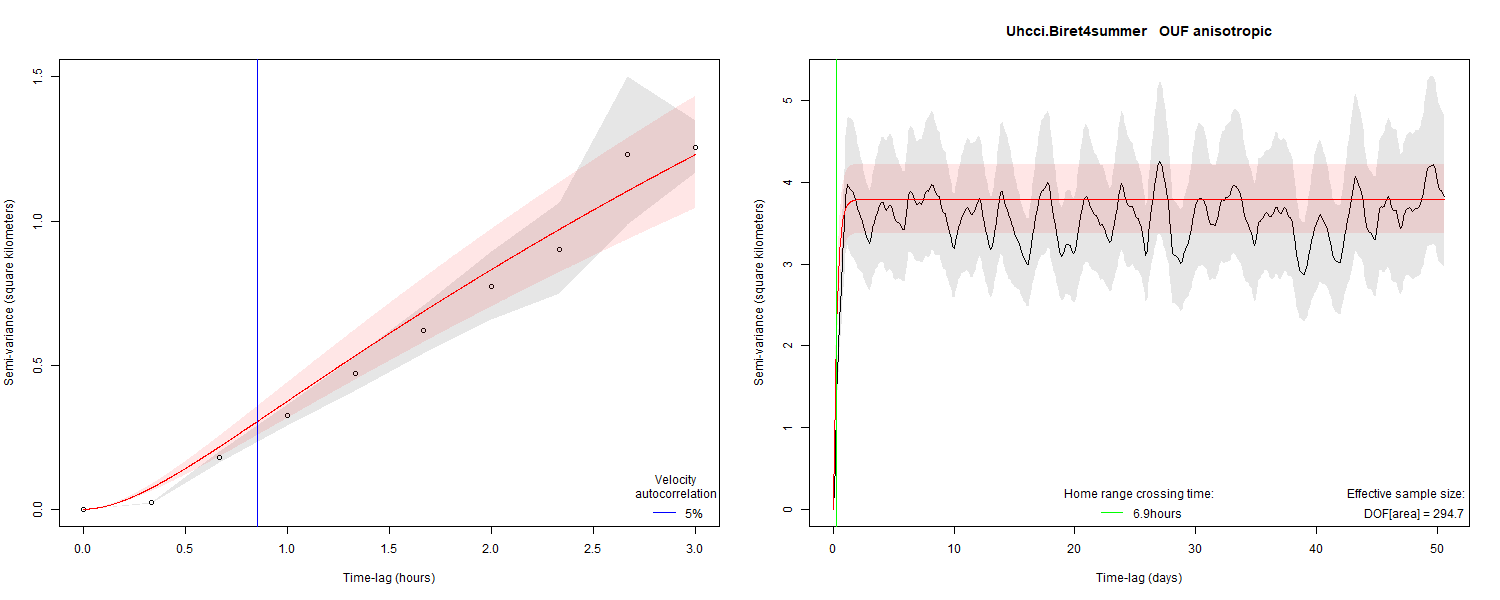

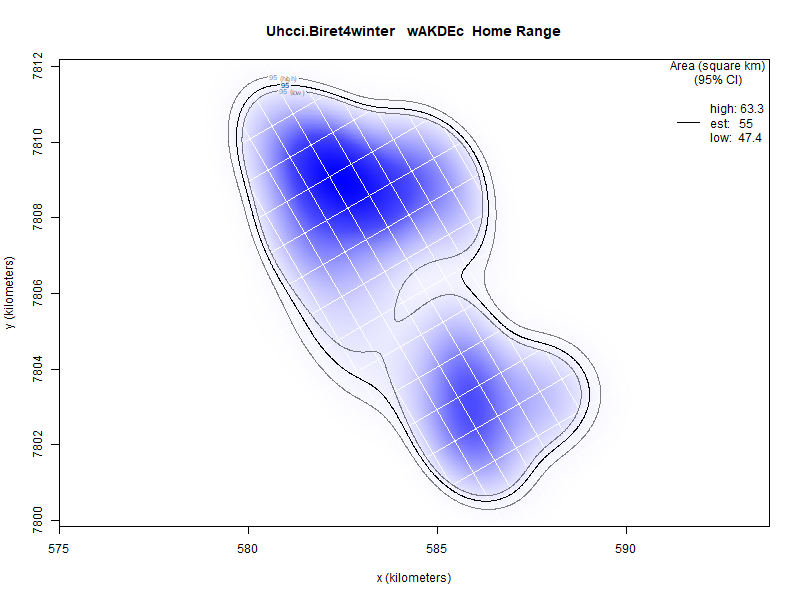

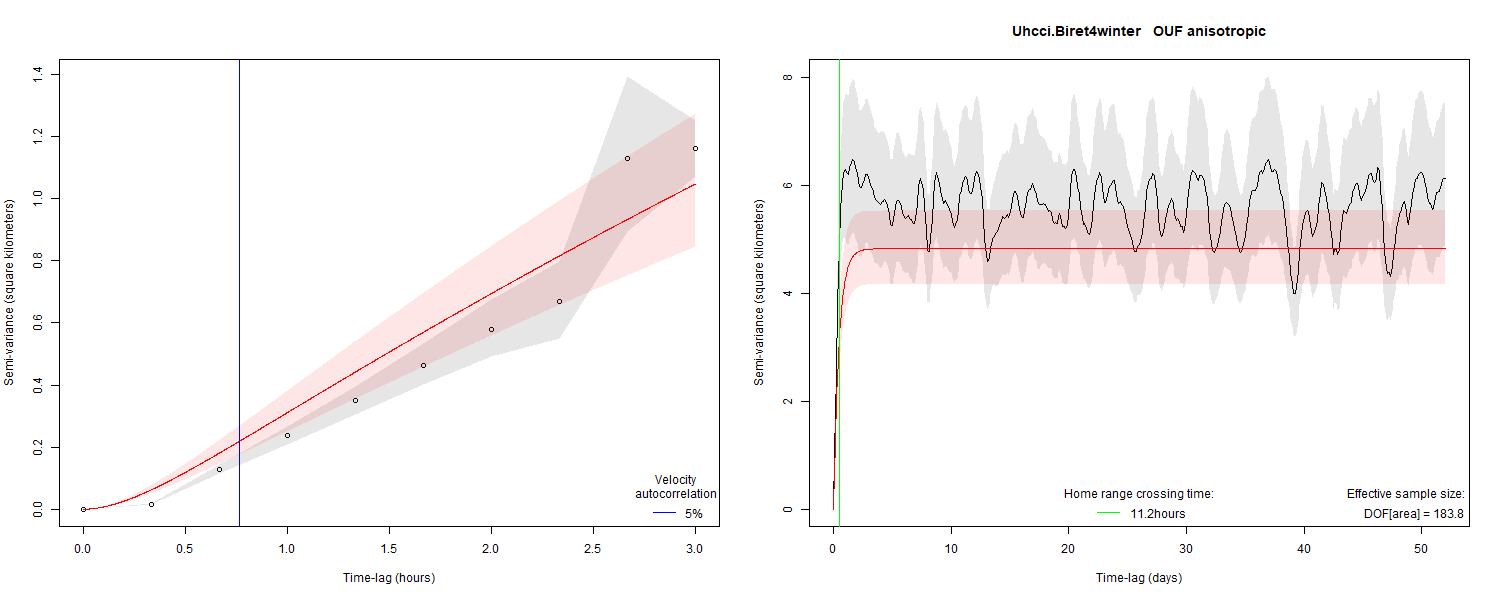

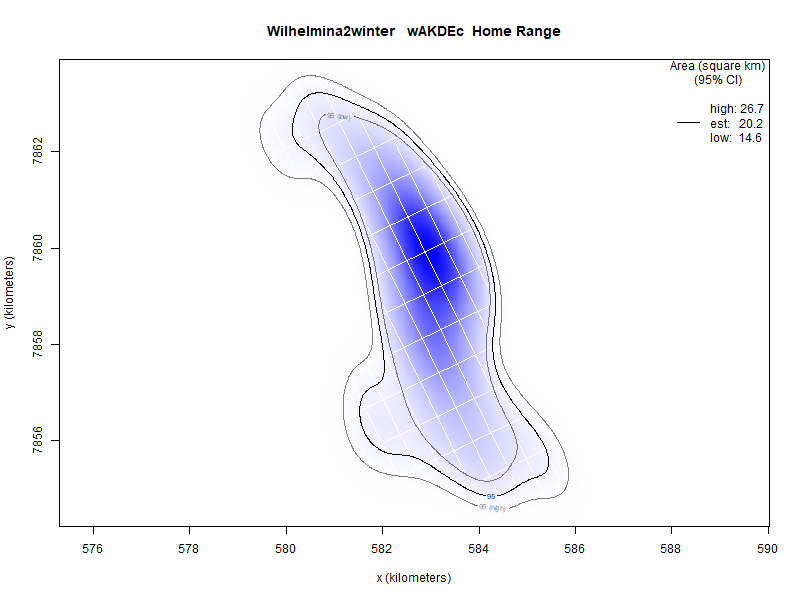

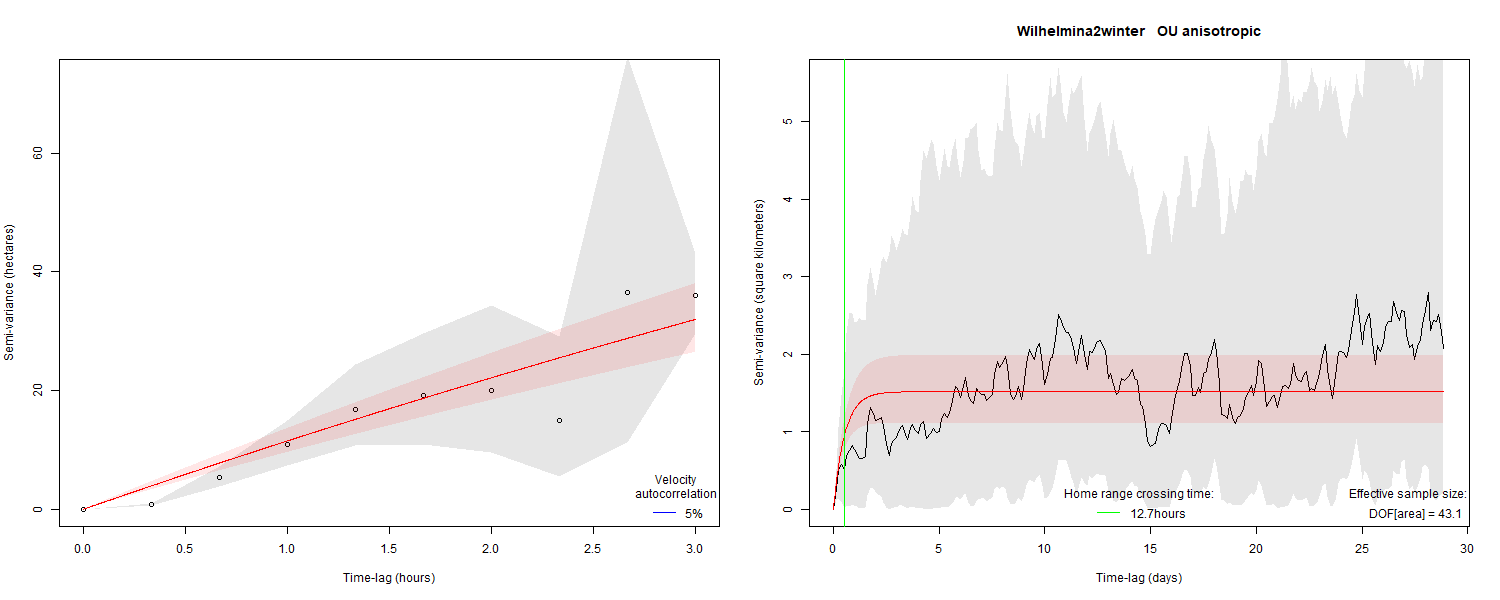

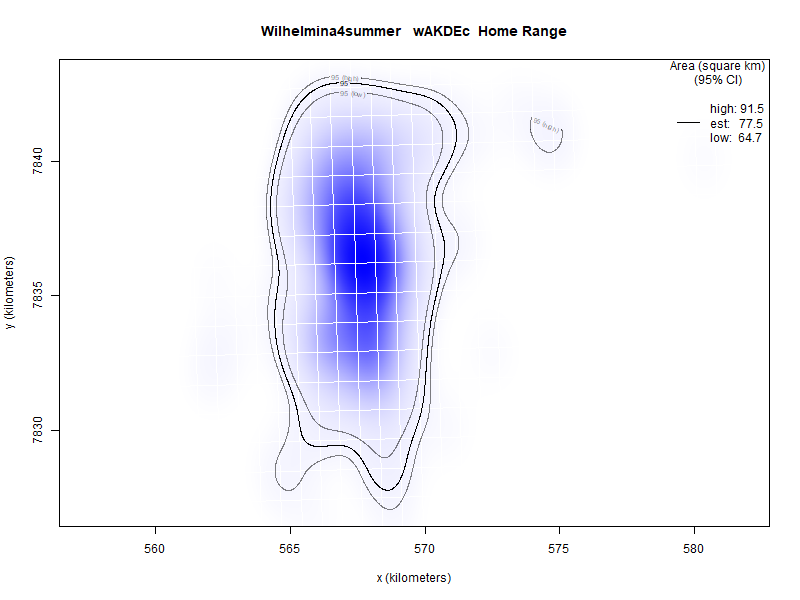

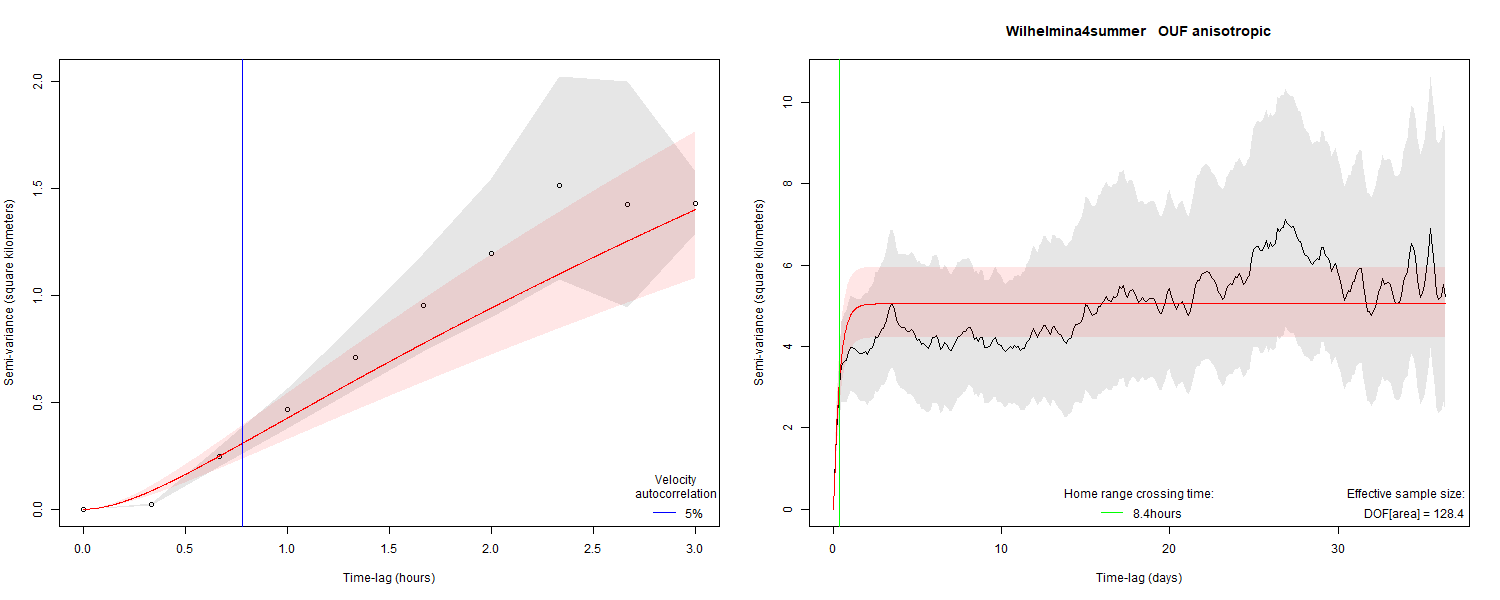

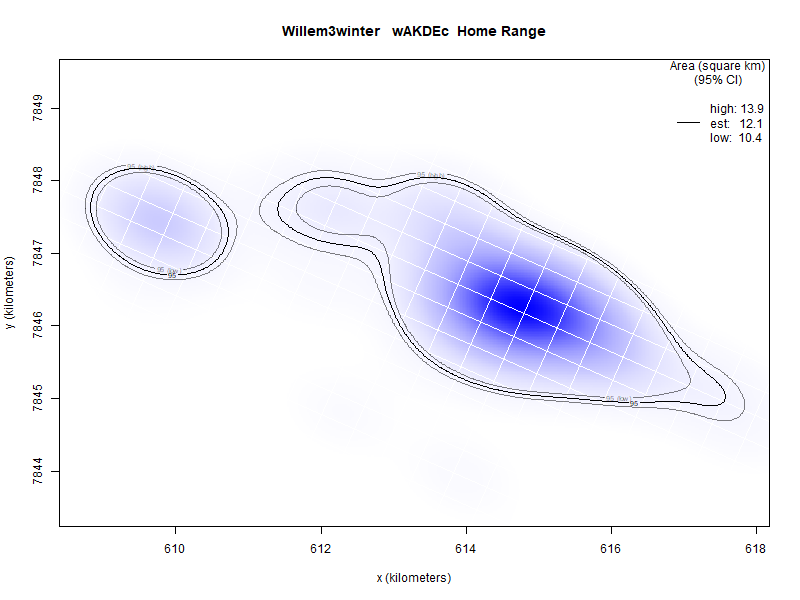

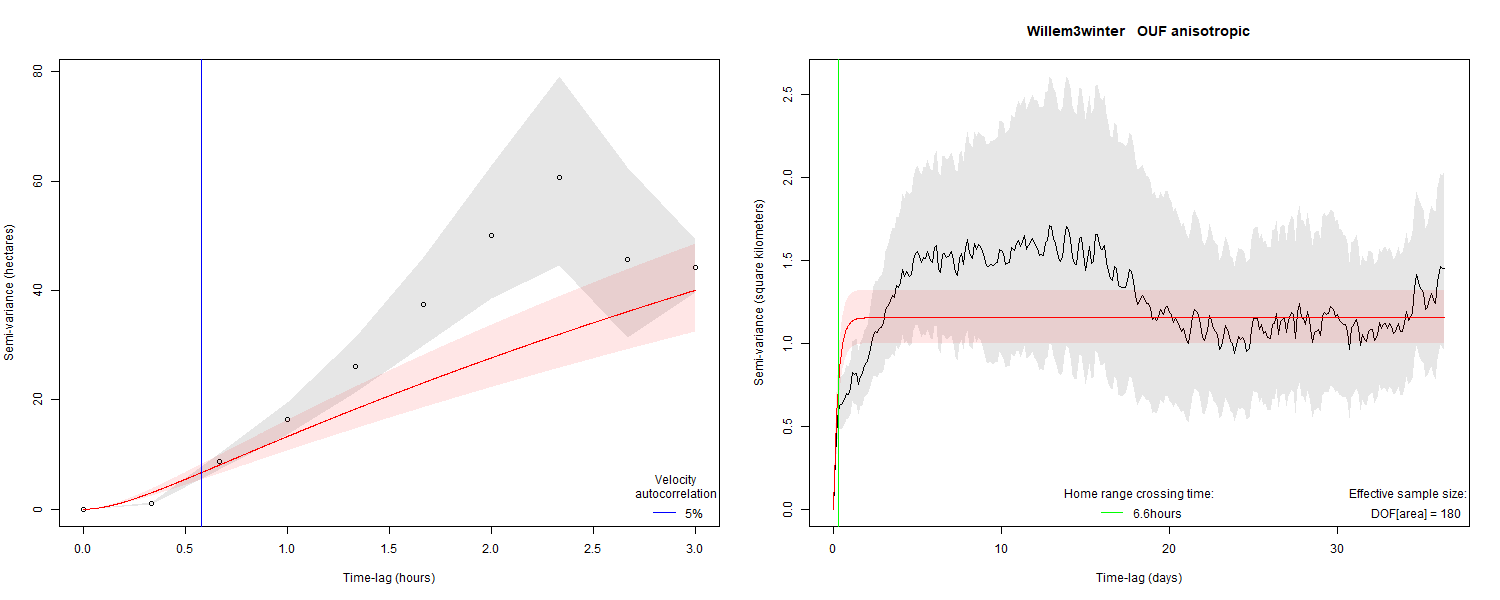

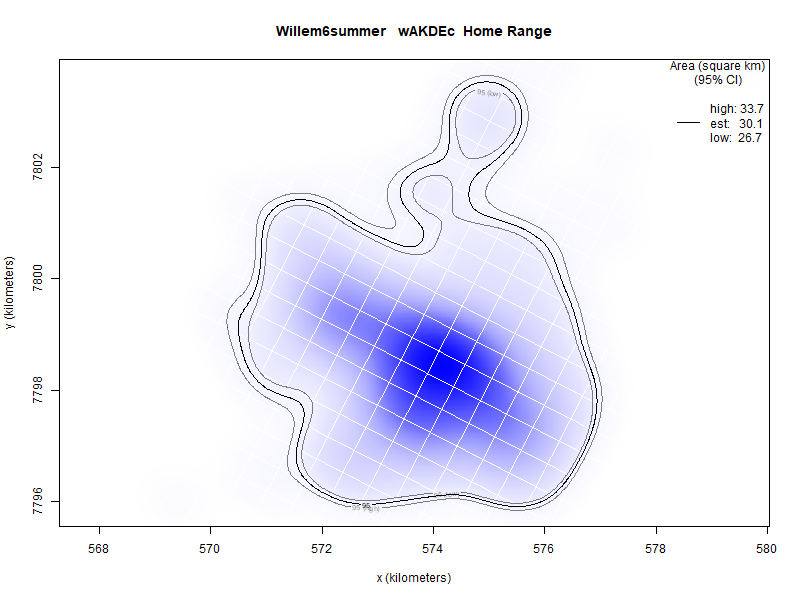

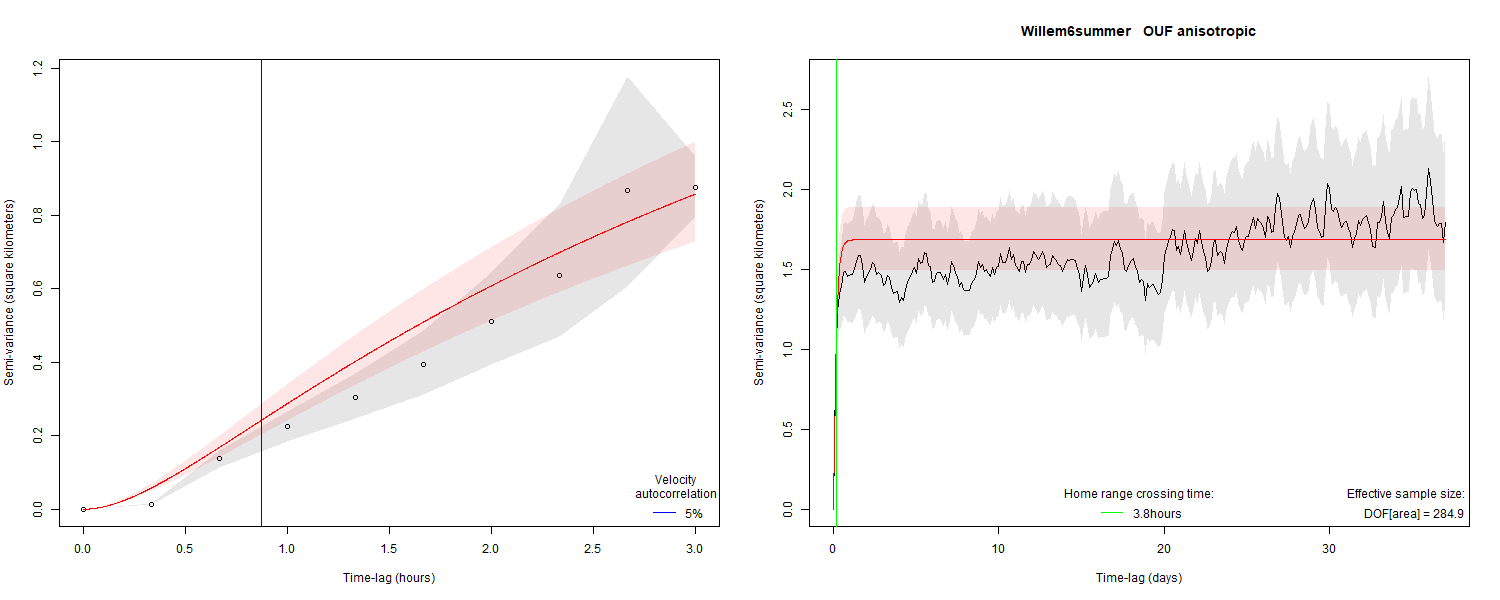

Supplement: Supplementary file 1 — Appendix S1: ece372512‐sup‐0001‐AppendixS1.docx. [file ECE3-15-e72512-s002.docx]
