# Supplementary material for: Space Use of an Expanding Generalist Predator Is Shaped by Human, Marine and Seasonal Effects on Arctic Tundra
Source: Ecol Evol. 2025 Nov 17;15(11):e72512. doi: 10.1002/ece3.72512 (PMC12620848; doi:10.1002/ece3.72512)
Supplement: Supplementary file 2 — Appendix S2: ece372512‐sup‐0002‐AppendixS2.docx. [file ECE3-15-e72512-s001.docx]

Appendix 2

*Home range size estimates per season, including additional information on underlying movement models and individual characteristics.*

| **Animal ID** | **Season** | **Home range size (95% CI)** | **Start date** | **End date** | **Duration (days)** | **Effective sample size (DOF)** | **Movement model** | **Life stage** | **Sex** | **Breeding status** | **Date of death** |
| --- | --- | --- | --- | --- | --- | --- | --- | --- | --- | --- | --- |
| Thorsen | Summer | 3.7 (3.3 - 4.0) | 19.05.2021 | 06.08.2021 | 79 | 450.9 | OUF anisotropic | adult | Male | unknown | 20.11.2022 |
| Thorsen | Winter | 30.8 (27.3 - 34.5) | 01.11.2021 | 01.03.2022 | 120 | 278 | OUF anisotropic | adult | Male | unknown | 20.11.2022 |
| Thorsen | Summer | 18.3 (16.5 - 20.3) | 15.05.2022 | 31.08.2022 | 108 | 357.7 | OUF anisotropic | adult | Male | unknown | 20.11.2022 |
| Reinaert | Winter | 10.5 (8.7 - 12.4) | 04.11.2021 | 14.12.2021 | 40 | 126.7 | OUF anisotropic | subadult | Male | unknown | 27.01.2022 |
| Kaptein Jan | Winter | 31.2 (27.1 - 35.6) | 05.11.2021 | 13.01.2022 | 69 | 207.6 | OUF anisotropic | subadult | Male | unknown | 05.11.2023 |
| Kaptein Jan | Summer | 80.1 (69.6 - 91.4) | 15.05.2022 | 10.08.2022 | 87 | 206.1 | OUF anisotropic | adult | Male | unknown | 05.11.2023 |
| Kaptein Jan | Winter | 129.0 (109.3 - 150.3) | 12.12.2022 | 01.03.2023 | 79 | 151.8 | OUF anisotropic | adult | Male | unknown | 05.11.2023 |
| Mari | Winter | 14.1 (12.8 - 15.4) | 05.11.2021 | 25.02.2022 | 112 | 460.7 | OUF anisotropic | subadult | Female | placental scars at death | 06.08.2022 |
| Mari | Summer | 10.2 (9.2 - 11.2) | 05.06.2022 | 04.08.2022 | 60 | 401.4 | OUF anisotropic | adult | Female | placental scars at death | 06.08.2022 |
| Uhcci Biret | Summer | 60.2 (53.5 - 67.2) | 06.06.2022 | 15.09.2022 | 101 | 294.7 | OUF anisotropic | adult | Female | unknown | 21.02.2023 |
| Uhcci Biret | Winter | 55.0 (47.4 - 63.3) | 01.11.2022 | 12.02.2023 | 103 | 183.8 | OUF anisotropic | adult | Female | unknown | 21.02.2023 |
| Murphy | Winter | 58.5 (47.6 - 70.6) | 09.11.2022 | 14.01.2023 | 66 | 98.8 | OUF anisotropic | subadult | Male | unknown | NA |
| Murphy | Summer | 9.5 (8.8 - 10.2) | 15.05.2023 | 15.09.2023 | 123 | 706.1 | OUF anisotropic | adult | Male | unknown | NA |
| Murphy | Winter | 13.3 (11.4 - 15.3) | 01.11.2023 | 29.02.2024 | 120 | 172.8 | OUF anisotropic | adult | Male | unknown | NA |
| Kate | Winter | 46.6 (36.8 - 57.7) | 08.12.2023 | 29.02.2024 | 83 | 76.2 | OUF anisotropic | adult | Female | placental scars at death | 05.01.2025 |
| Pedersen | Winter | 29.1 (24.5 - 34.1) | 14.01.2024 | 12.02.2024 | 29 | 139.8 | OUF anisotropic | subadult | Female | unknown | NA |
| Wilhelmina | Winter | 20.2 (14.6 - 26.7) | 17.11.2024 | 14.01.2025 | 58 | 43.1 | OU Anisotropic | adult | Female | unknown | NA |
| Wilhelmina | Summer | 77.5 (64.7 - 91.5) | 22.05.2025 | 03.08.2025 | 73 | 128.4 | OUF anisotropic | adult | Female | unknown | NA |
| Willem | Winter | 12.1 (10.4 - 13.9) | 08.12.2024 | 19.02.2025 | 73 | 180 | OUF anisotropic | subadult | Male | unknown | NA |
| Willem | Summer | 30.1 (26.7 - 33.7) | 22.05.2025 | 04.08.2025 | 74 | 284.9 | OUF anisotropic | adult | Male | unknown | NA |
| Tina | Winter | 13.7 (11.8 - 15.6) | 15.12.2024 | 22.02.2025 | 69 | 195 | OU Anisotropic | subadult | Female | unknown | NA |
| Sarre | Winter | 17.8 (16.1 - 19.6) | 21.11.2024 | 01.03.2025 | 100 | 400.1 | OUF anisotropic | subadult | Male | unknown | NA |
